# Ligase 1 is a predictor of platinum resistance and its blockade is synthetically lethal in XRCC1 deficient epithelial ovarian cancers

**DOI:** 10.7150/thno.51456

**Published:** 2021-07-25

**Authors:** Reem Ali, Muslim Alabdullah, Mashael Algethami, Adel Alblihy, Islam Miligy, Ahmed Shoqafi, Katia A. Mesquita, Tarek Abdel-Fatah, Stephen YT Chan, Pei Wen Chiang, Nigel P Mongan, Emad A Rakha, Alan E Tomkinson, Srinivasan Madhusudan

**Affiliations:** 1 Nottingham Biodiscovery Institute, School of Medicine, University of Nottingham, University Park, Nottingham NG7 3RD, UK.; 2 Department of Pathology, Division of Cancer and Stem Cells, School of Medicine, University of Nottingham, Nottingham NG51PB, UK.; 3 Medical Center, King Fahad Security College (KFSC), Riyadh 11461, Saudi Arabia.; 4 Department of Oncology, Nottingham University Hospitals, City Hospital Campus, Nottingham NG5 1PB, UK.; 5 Department of Obstetrics & Gynaecology, Queens Medical Centre, Nottingham University Hospitals, Nottingham NG7 2UH, UK.; 6 Faculty of Medicine and Health Sciences, Centre for Cancer Sciences, University of Nottingham, Sutton Bonington Campus, Sutton Bonington, Leicestershire LE12 5RD, UK; 7 Department of Pharmacology, Weill Cornell Medicine, New York, NY, 10065, USA; 8 Department of Internal Medicine, Division of Molecular Medicine, Health Sciences Center, The University of New Mexico, Albuquerque, NM 87102, USA.

**Keywords:** Ovarian cancer, DNA repair, LIG1, LIG3, LIG4, LIG1 inhibitor, Prognostics, Predictive bimarker, Synthetic lethality

## Abstract

**Rationale:** The human ligases (LIG1, LIG3 and LIG4) are essential for the maintenance of genomic integrity by catalysing the formation of phosphodiester bonds between adjacent 5′-phosphoryl and 3′-hydroxyl termini at single and double strand breaks in duplex DNA molecules generated either directly by DNA damage or during replication, recombination, and DNA repair. Whether LIG1, LIG3 and LIG4 can influence ovarian cancer pathogenesis and therapeutics is largely unknown.

**Methods:** We investigated LIG1, LIG3 and LIG4 expression in clinical cohorts of epithelial ovarian cancers [protein level (n=525) and transcriptional level (n=1075)] and correlated to clinicopathological features and survival outcomes. Pre-clinically, platinum sensitivity was investigated in LIG1 depleted ovarian cancer cells. A small molecule inhibitor of LIG1 (L82) was tested for synthetic lethality application in XRCC1, BRCA2 or ATM deficient cancer cells.

**Results:** LIG1 and LIG3 overexpression linked with aggressive phenotypes, platinum resistance and poor progression free survival (PFS). In contrast, LIG4 deficiency was associated with platinum resistance and worse PFS. In a multivariate analysis, LIG1 was independently associated with adverse outcome. In ovarian cancer cell lines, LIG1 depletion increased platinum cytotoxicity. L82 monotherapy was synthetically lethal in XRCC1 deficient ovarian cancer cells and 3D-spheroids. Increased cytotoxicity was linked with accumulation of DNA double strand breaks (DSBs), S-phase cell cycle arrest and increased apoptotic cells. L82 was also selectively toxic in BRCA2 deficient or ATM deficient cancer cells and 3D-spheroids.

**Conclusions:** We provide evidence that LIG1 is an attractive target for personalization of ovarian cancer therapy.

## Introduction

PARP inhibitor (Niraparib, Olaparib, Rucaparib) maintenance therapy improves progression free survival in BRCA germ-line deficient and platinum sensitive sporadic epithelial ovarian cancers 1-3. However, PARP maintenance therapy is beneficial only in about 50% of patients. Intrinsic or acquired resistance to PARP inhibitors is a considerable clinical challenge 4, 5 and the development of alternative synthetic lethality approaches is urgently required.

DNA ligases encoded by the human genes *LIG1, LIG3,* and* LIG4* are nucleotidyl transferases (NTases) that catalyse phosphodiester bond formation in an ATP-dependent mechanism 6. These enzymes, which share a related catalytic core composed of an NTase domain, OB-fold domain and an N-terminal DNA-binding domain (DBD) 7, have multiple overlapping roles in nuclear replication, recombination and DNA repair 7-9. In contrast, DNA joining function in mitochondrial DNA metabolism is due to a single DNA ligase species encoded by the *LIG3* gene 8, 10-13.

During DNA replication, DNA ligase I (LIG1) is the major enzyme joining Okazaki fragments although it appears that DNA ligase IIIα (LIG3α) is able to fulfil this role in LIG1-deficient cells. There is also functionally redundancy between these enzymes in excision repair, single strand break repair and alternative end joining 9. The participation of LIG1 in DNA replication and repair is mediated by protein-protein interactions with different partners, proliferating cell nuclear antigen (PCNA), replication factor C, hRad9-hRad1-hHus1 (9-1-1) complex and DNA polymerase β that mostly involve the non-catalytic N-terminal region of LIG1 8. In addition to its catalytic core, LIG3α has an N-terminal zinc-finger domain which serves as a nick sensor and a C-terminal BRCT (breast and ovarian cancer susceptibility protein 1) domain that stably interacts with XRCC1, a key scaffolding protein that binds to multiple DNA repair enzymes. DNA ligase IV (LIG4) also functions in a stable complex with a DNA repair protein, XRCC4, but, unlike the other nuclear DNA ligases, its role is limited to the repair of DNA double strand breaks by non-homologous end joining (NHEJ) together with the DNA dependent protein kinase and XLF.

Inherited human LIG1- and LIG4- deficiency syndromes have been described. The symptoms associated with LIG1 deficiency include retarded growth and development as well as an unexplained immunodeficiency. At the cellular level, LIG1-deficiency results in abnormal Okazaki fragment processing and hypersensitivity to DNA alkylating agents. Human LIG4 deficiency also causes immunodeficiency but here it is due the role of LIG4 in immunoglobulin gene rearrangement as well as a predisposition to cancer 14. LIG4 deficient cells are sensitive to ionizing radiation due to a defect in the repair of DSBs by NHEJ 15. To date, no LIG3α-deficient individuals have been identified, presumably because of the essential, unique function of LIG3α in mitochondrial DNA metabolism.

Altered expression of the human DNA ligases has been observed in cancers. While elevated expression of LIG1 is frequently observed in cancer cell lines, this has been assumed to reflect the hyperproliferative state of cancer cells since LIG1 expression correlates with proliferation 6. Abnormal Wnt signalling results in increased expression of LIG4, conferring radioresistance in Wnt-driven cancers. Conversely, cancer cells with reduced expression of LIG4 and a reciprocal increase in LIG3α expression exhibit an increased dependence on alternative end-joining for DSB repair and sensitivity to PARP and LIG3 inhibitors 8. Given the key roles played by LIG1, LIG3 and LIG4 in genomic integrity 8 and the response of cancer cells to therapy, we investigated their role in ovarian cancer pathology and potential as novel therapeutic targets.

## Materials and Methods

Full details are available in Supplementary material and methods.

### Clinical study

LIG1, LIG3, LIG4, XRCC1 immunohistochemistry was completed in 525 patients with histologically confirmed ovarian cancer and treated from 1997 to 2010 at Nottingham University Hospitals (NUH). This study was carried out in accordance with the declaration of The Helsinki and ethical approval which was obtained from the Nottingham Research Ethics Committee (REC Approval Number 06/Q240/153). All patients provided written informed consent. See supplementary methods for full details.

Predictive and prognostic significance of *LIG1, LIG3 and LIG4* mRNA expression mRNA expression was investigated in publicly available ovarian tumour gene expression data sets (http://kmplot.com/analysis/index.php?p=service&cancer=ovar)^16^. A total of 1075 Serous cystadenocarcinomas were included in this analysis.

### Pre-clinical study

A2780, A2780cis, PEO1 and PEO4 were purchased from American Type Culture Collection (ATCC, Manassas, USA). XRCC1-deficient HeLa SilenciX cells, BRCA2-deficient HeLa SilenciX cells, ATM-deficient HeLa SilenciX cells and controls XRCC1, BRCA2 or ATM -proficient HeLa SilenciX cells were purchased from Tebu-Bio (www.tebu-bio.com).

Methodology for transient knockdown of LIG1 and generation of XRCC1 knock out using CRISPR-cas9 system are described in supplementary methods.

Compounds, reagents, clonogenic assays, cell proliferation assays, confocal microscopy, immunoprecipitation, functional assays (FACS, cell cycle progression, apoptosis assays), 3D-spheroid assays, exome sequencing and bioinformatics are described in supplementary methods.

## Results

**LIG1, LIG3 and LIG4 expression profiling in epithelial ovarian cancers:** We initially evaluated LIG1, LIG3 and LIG4 expression at protein and transcriptional level in clinical cohorts of epithelial ovarian cancers. Patients' demographics are summarized in Table S1.

**Figure 1:**
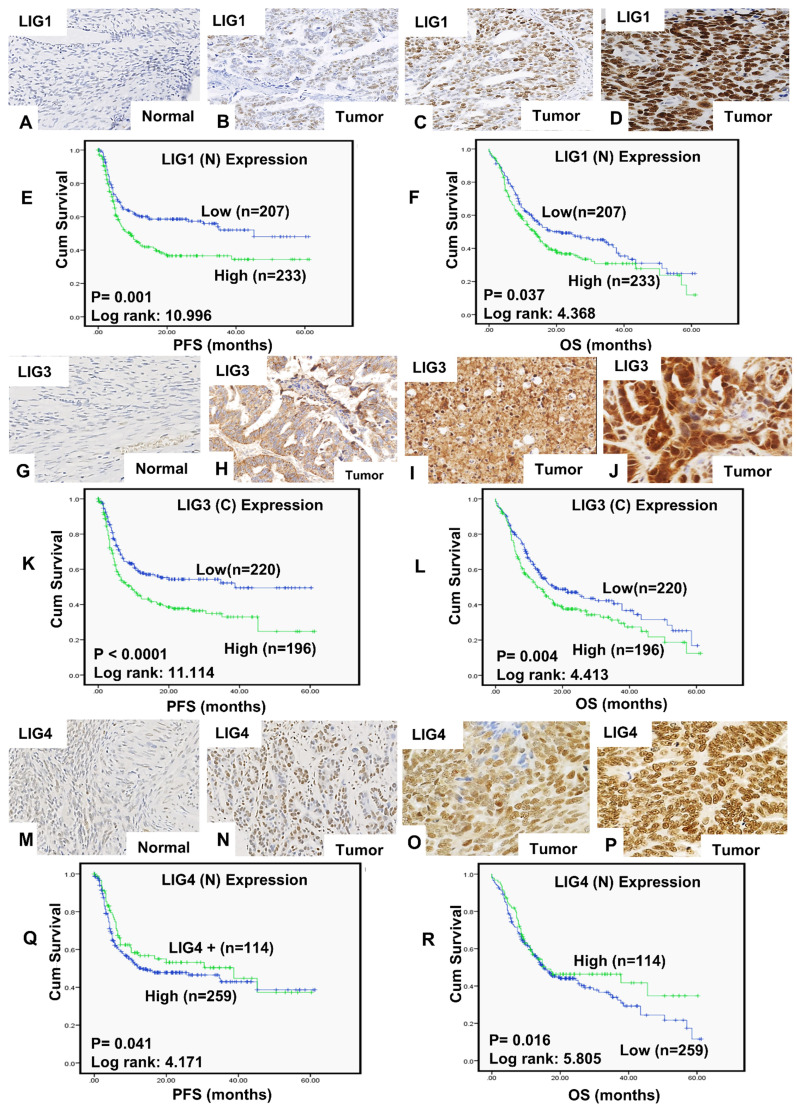
Expression of LIG1by immunohistochemistry in tissue microarray images. **A**) Negative LIG1 staining in normal ovarian tissue, **B**) weak nuclear LIG1 staining in tumor, **C**) moderate nuclear LIG1 staining staining in tumor, **D**) strong nuclear LIG1 staining staining in tumor. All images were captured at 20-times magnifications. (**E**)Kaplan Meier curves for LIG1 nuclear expression and progression free survival (PFS). (**F**) Kaplan Meier curves for LIG1nuclear expression and overall survival (OS). Expression of LIG3 by immunohistochemistry in tissue microarray images. **G**) Negative LIG3 staining in normal ovarian tissue, **H**) weak LIG3 staining in tumor, **I**) moderate LIG3 in tumor, **J**) strong LIG3 staining in tumor**.** All images were captured at 20-times magnifications. (**K**) Kaplan Meier curves for LIG3 cytoplasmic expression alone and progression free survival (PFS). (**L**) Kaplan Meier curves for LIG3 cytoplasmic expression alone and overall survival (OS). Expression of LIG4 by immunohistochemistry in tissue microarray images.** M**) Negative LIG4 staining in normal ovarian tissue, **N**) weak LIG4 staining in tumor, **O**) moderate LIG4 staining in tumor and **P**) strong LIG4 staining in tumor. All images were captured at 20-times magnifications. (**Q**) Kaplan Meier curves for LIG4 nuclear expression and progression free survival (PFS). (**R**) Kaplan Meier curves for LIG4 nuclear expression and overall survival (OS). All p-values were generated by log-rank.

A total of 442 tumours were suitable for analysis of LIG1 nuclear expression. We did not observe any cytoplasmic staining for LIG1 (Figure 1A-D). 207/442 (46.8%) tumours were low for LIG1 expression and 234 (53.2%) of the tumours were high in expression. High nuclear LIG1 was significantly associated with serous carcinoma (p < 0.0001), higher FIGO stage at presentation (p < 0.0001), higher tumour grade (p < 0.0001), sub-optimal debulking (p = 0.004) and residual tumour following surgery (p = 0.014) compared to tumours with low LIG1 expression. Tumours with high LIG1 nuclear expression were likely to be platinum resistance although this was non-significant (p = 0.063) (Table 1). Patients whose tumours had high LIG1 nuclear expression had significantly poorer progression free survival (PFS) (p = 0.001) (Figure 1E) and overall survival (OS) (p = 0.037) (Figure 1F) compared patients with low LIG1 nuclear expressing tumours. At the transcriptional level, similarly, high *LIG1* mRNA was associated with poor PFS (p = 0.029) (Figure S1A) and OS (p = 0.019) (Figure S1B).

A total of 418 tumours were suitable for analysis of LIG3 expression. We observed both cytoplasmic and nuclear expression of LIG3 (Figure 1G-J). Low cytoplasmic LIG3 was seen in 221/418 (52.9%) tumours and high cytoplasmic LIG3 was observed in 197/418 (47.1%) of the tumours. Low nuclear LIG3 was seen in 219/418 (52.4%) of the tumours and 199/418 (47.6%) of tumours had high nuclear expression. We initially investigated cytoplasmic or nuclear expression individually. High cytoplasmic expression was significantly associated with higher FIGO stage of cancer (p = 0.002), higher histology grade (p = 0.028), residual tumour following surgery (p = 0.001), measurable disease before chemotherapy (p= 0.006), platinum resistance (p = 0.025) (Table S2), PFS (p < 0.0001) (Figure 1K) and OS (p < 0.00001) (Figure 1L). High nuclear staining was significantly associated with serous type carcinoma (p = 0.017) (Table S3). Nuclear LIG3 expression did not significantly influence PFS (p = 0.418) (Figure S1C) or OS (p = 0.450) (Figure S1D). When cytoplasmic and nuclear expression was combined, we observed that tumours with high cytoplasmic/low nuclear LIG3 co-expression had worse PFS (Figure S2A) and worse OS (Figure S2B) compared to tumours with low cytoplasmic/low nuclear LIG3 co-expression. At the transcriptional level, high *LIG3* mRNA was associated with significantly poor PFS (p = 0.023) (Figure S2C) but not with OS (p = 0.11) (Figure S2D).

A total of 374 tumours were suitable for analysis of LIG4 nuclear expression. We did not observe any cytoplasmic staining for LIG4 (Figure 1M-P). Low nuclear LIG4 was seen in 260/374 (69.5%) tumours and 114/374 (30.5%) of the tumours had high LIG4 nuclear expression. Low nuclear LIG4 was significantly associated with larger residual tumours (p = 0.006), poor response to platinum-based chemotherapy (p = 0.049) (Table S4), poor PFS (p = 0.041) (Figure 1Q) and poor OS (p = 0.016) (Figure 1R) compared with high nuclear LIG4 expressing tumours. At the transcriptional level, low *LIG4* mRNA was non-significant for poor PFS (p = 0.091) (Figure S3A) and OS (p = 0.26) (Figure S3B).

**Multivariate analysis:** LIG1, LIG3 and LIG4 protein expressions were investigated in a Cox multivariate model (Table S5). High nuclear LIG1 expression remained independently associated with poor PFS (p = 0.001) as well as poor OS (p = 0.029). Although high cytoplasmic LIG3 influenced PFS (p=0.045), it did not influence OS (p=0.167). LIG4 was not an independent predictive or prognostic marker.

Given the independent significance of LIG1, we also conducted sub-group analysis in platinum sensitive and resistant ovarian cancer (Figure S4). As shown in Figure S4A-B, LIG1 predicted PFS and associated with OS in the platinum sensitive group. We proceeded to pre-clinical evaluation of LIG1 in ovarian cancer cells.

**Figure 2:**
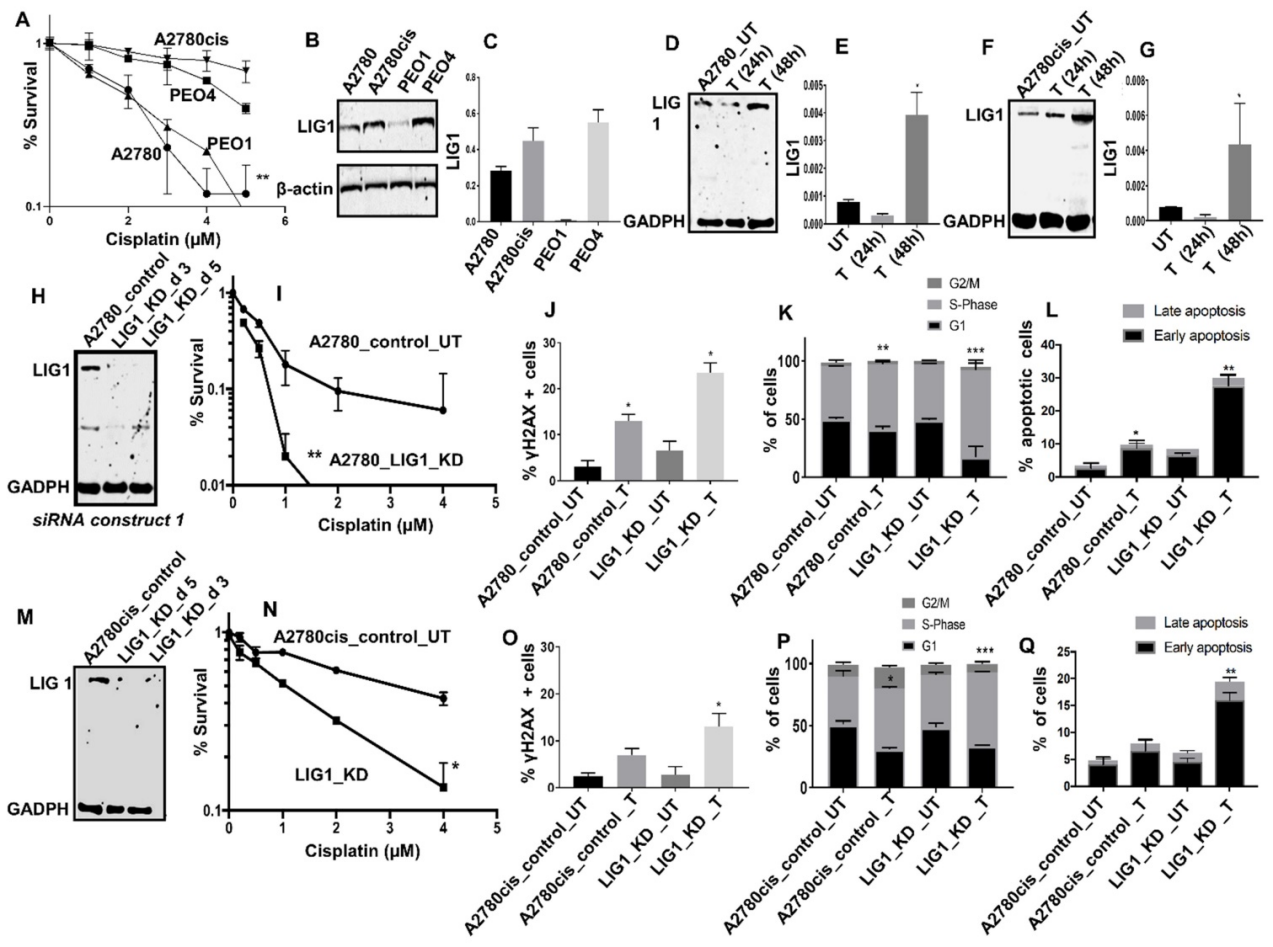
(**A**) Clonogenic survival assay for A2780, A2780cis, PEO1&PEO4 cells in different doses of cisplatin. (B) Western blot for LIG1 expression in A2780, A2780cis, PEO1&PEO4 cells. (C) Quantification of LIG1protein levels is shown here. (**D**) Western blot for LIG1 protein levels in A2780 cells treated with Cisplatin (5 μM) Lysates were collected at 24 and 48 hrs post treatment. (**E**) Quantification of LIG1 protein levels by western blot in A2780 cells treated with Cisplatin. (H) Western blot for LIG1 knockdown in A2780 cells. Cells were plated in T25 flasks overnight and transfected with scrambled control or LIG1 siRNA. Transfection efficiency was confirmed by western blotting at day3 and day 5. Figures are representative of 3 or more independent experiments. (**I**) Clonogenic survival assay for Cisplatin sensitivity in A2780 control and LIG1 knock down (p-value was calculated as an average across control and KD cell line). (**J**) Quantification of γH2AX positive cells by flow cytometry in A2780 cells control and LIG1_ knock down treated with 5µM cisplatin for 24 h. (**K**) Cell cycle analysis by flow cytometry in A2780 cells control and LIG1_ knockdown treated with5 μM cisplatin. (**L**) AnnexinV analysis for apoptotic cells in A2780 cells control and LIG1_ knock down treated with5 μM cisplatin. (**M**) LIG1 knock down by siRNA in A2780cis cells. (**N**) Clonogenic survival assay for Cisplatin sensitivity in A2780cis control and LIG1 knock down. (**O**) Western blot for LIG1 protein levels in A2780cis cells treated with Cisplatin (5 μM). Lysates were collected at 24 and 48 hrs post treatment. (**N**) Quantification of LIG1 protein levels by western blot in A2780cis cells treated with Cisplatin. (**O**) Quantification of γH2AX positive cells by flow cytometry in A2780cis cells control and LIG1_ knock down treated with 5µM cisplatin for 24 h. (**P**) Cell cycle analysis by flow cytometry in A2780cis cells control and LIG1_ knockdown treated with5 μM cisplatin. (**Q**) Annexin V analysis for apoptotic cells in A2780cis cells control and LIG1_ knock down treated with5 μM cisplatin. cells were seeded overnight transfected with scrambled control or LIG1 siRNA. At day 3 controls and knockdown cells were re platted in 6-well plates overnight and treated with 5 μM cisplatin and analyzed by flow cytometry on day 5. Figures are representative of 3 or more independent experiments. Error bars represents standard error of mean (SEM) between experiments. '*' = P - values < 0.05, '**' = P- values < 0.01.

**LIG1 depletion promotes platinum sensitivity:** We conducted pre-clinical studies of LIG1 in platinum sensitive (A2780, PEO1, Figure 2A) and platinum resistant (A2780cis, PEO4, Figure 2A) ovarian cancer cell lines. At baseline, LIG1 protein expression was high in A2780cis and PEO4 cells (Figure 2B-C). To evaluate if LIG1 expression is induced after cisplatin treatment, we generated whole cell extracts at baseline, 24 hours, and 48 hours of cisplatin therapy. As shown in Figure 2D-G, there was an increase in LIG1 levels at 48 hours in both A2780 and A2780cis cell lines compared to untreated cells (Figure 2D-G).

The data suggests LIG1 may influence platinum sensitivity and is induced after platinum therapy in ovarian cancer cells. In A2780 cells, when LIG1 was transiently depleted using siRNA (Figure 2H), we observed substantial sensitization to platinum therapy compared to scrambled controls as evaluated by clonogenic assays (Figure 2I). Increased sensitivity was associated with accumulation of DNA double strand breaks (DSB) (Figure 2J, Figure S5A-D), increased p-ATM & p-Chk1 level (Figure S6A-D**),** S-phase cell cycle arrest (Figure 2K, Figure S5E-H) and increased apoptotic cells (Figure 2L, Figure S5I-L). We then validated using another siRNA construct. As shown in Figure S6E-F, LIG1 depletion increased platinum sensitivity compared to scrambled control. To evaluate whether LIG1 depletion could reverse platinum resistance we depleted LIG1 A2780cis cells (Figure 2M) and observed substantial re-sensitization to platinum therapy (Figure 2N) which was also associated with DSB accumulation (Figure 2O), increased p-ATM & p-Chk1 level (Figure S6B-D**),** S-phase cell cycle arrest (Figure 2P) and increased apoptotic cells (Figure 2Q). The data provides evidence that LIG1 depletion not only enhances platinum sensitivity but can also reverse platinum resistance in ovarian cancer cells. Taken together, the pre-clinical and clinical data provides evidence that LIG1 is a predictor of response to platinum therapy in ovarian cancers.

**Figure 3:**
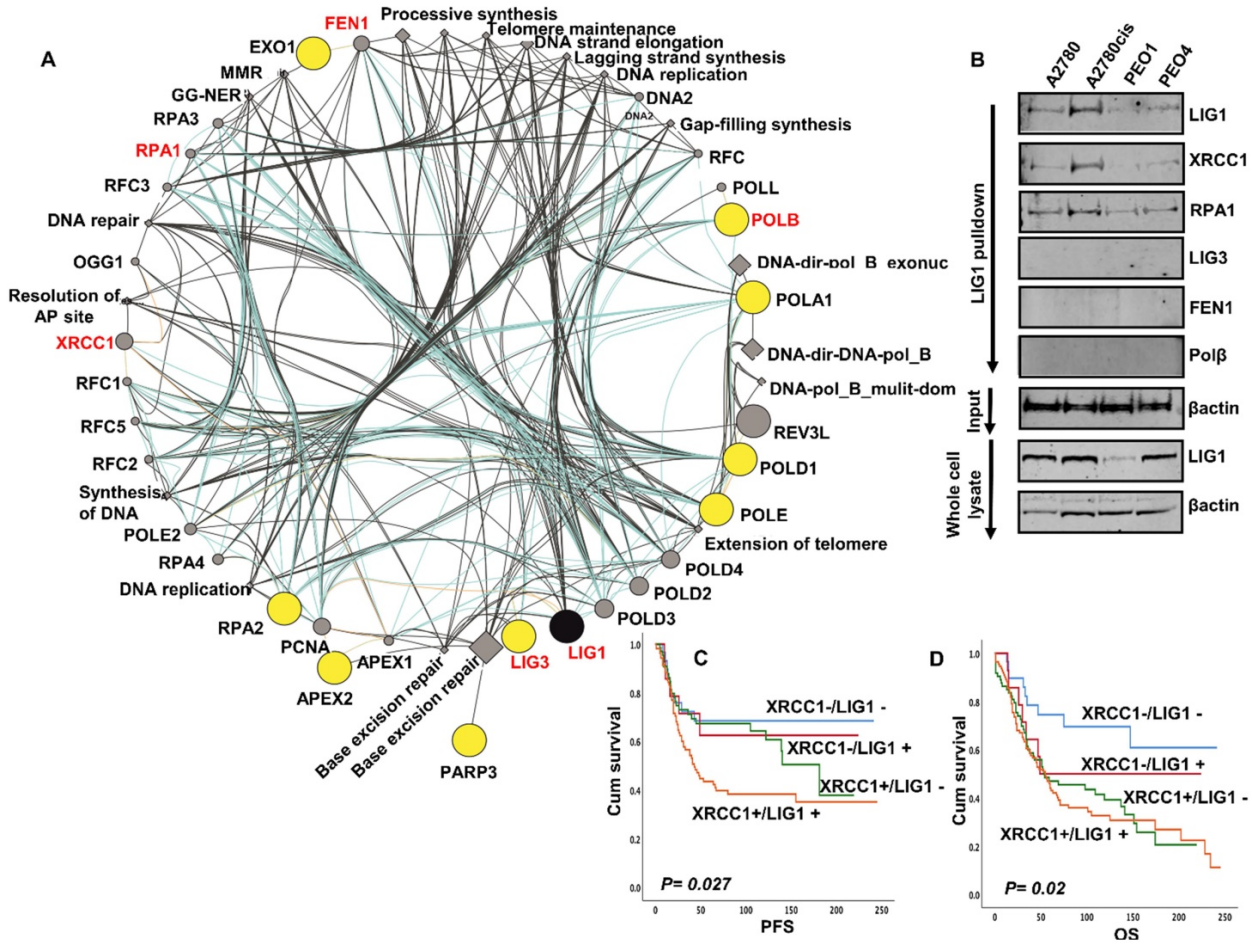
(**A**) LIG1 is involved in DNA replication (has03030), base excision repair (hsa03410), nucleotide excision repair (hsa03420) and mismatch repair (hsa03430). We assessed the mutation status of all 93 genes involved in these pathways (Supplemental Table S6). Coding variants including frameshift, stop gain and substitution variants were identified in the genes encoding APEX2, EXO1, LIG3, PARP3, POLA1, POLB, POLD1, POLE, RPA2. The Genemania plugin for Cytoscape was used to generate a pathway map identifying LIG1 and its functionally associated genes which harbor coding variants in Pt resistant A2780cis and PE04 cell lines. The protein nodes indicated in yellow cicles are those LIG1 interactors with variants associated with Pt resistance in these cell lines. No variants were identified in those variants shaded in grey circles. All nodes are scaled to indicated connectedness, that it is to say the number of interactions identified. Inferred pathways are indicated as grey diamond. (**B**) LIG1 co-immunoprecipitation with XRCC1 and RPA in A2780, A2780cis,PEO1. (**C**) Kaplan Meier curves for LIG1 & XRCC1 co-expression and PFS. (**D**) Kaplan Meier curves for LIG1 & XRCC1 co-expression and OS.

**Bioinformatics analysis of the LIG1 interactome:** Inherited human LIG1 deficiency syndromes have been described. We performed next generation exome sequencing (NGS) in A2780, A2780cis, PEO1 and PEO4. The full NGS data has been uploaded and is available at https://www.ncbi.nlm.nih.gov/sra/PRJNA731652. No coding variants of *LIG1* were identified in platinum sensitive A2780 & PE01, or in A2780cis & PE04 platinum resistant derivatives. We next assessed the mutation status of all genes ascribed to LIG1 associated DNA replication (has03030), base excision repair (hsa03410), nucleotide excision repair (hsa03420) and mismatch repair (hsa03430) KEGG pathways. Of the 93 genes involved in these LIG1-associated pathways (Table S6), coding variants including frameshift, stop gain and substitution variants were identified in the genes encoding APEX2, EXO1, LIG3, PARP3, POLA1, POLB, POLD1, POLE, RPA2 (Figure 3A).

To evaluate for any physical interaction between LIG1 and a panel DNA repair proteins (LIG3, POLB, FEN1, RPA, XRCC1) involved in the LIG1 associated pathways (Figure 3A), we conducted co-immunoprecipitation studies. Cell lysates were first incubated with LIG1 antibody, then conjugated to protein A /G magnetic beads, washed, eluted and western blotted for LIG3, POLB, FEN1, RPA and XRCC1 detection. As shown in Figure 3B, we observed that LIG1 physically associated with RPA1, FEN1 and XRCC1. Moreover, the expression of FEN1, RPA1 and XRCC1 was higher in A2780cis and PEO4 lysates compared to A2780 and PEO1 lysates. We have previously shown that FEN1 17, RPA1 (manuscript under preparation) and XRCC1 18 are key predictor of platinum resistance in ovarian cancers. The data suggest that the LIG1 functional interactome may contribute to platinum resistance either directly or indirectly through interactions with other factors, such as XRCC1, involved in processing platinum induced DNA damage. We evaluated the clinical significance of XRCC1 and LIG1 protein co-expression in human ovarian cancers. As shown in Figure 3C, patients whose tumours had high LIG1/high XRCC1 had worse PFS after platinum-based chemotherapy compared to patients whose tumours had low LIG1/low XRCC1 co-expression. Similarly, OS was poor in patients whose tumours had high LIG1/high XRCC1 compared to patients whose tumours had low LIG1/low XRCC1 co-expression (Figure 3D). Taken together, the data implies that LIG1 blockade by small molecule inhibitor could be a promising strategy in XRCC1 deficient or proficient ovarian cancers.

**LIG1 blockade is synthetically lethal in XRCC1 deficient cancer cells:** A** s**mall molecule inhibitor targeting LIG1 was generated as described previously 6. Briefly, computer-aided drug design was used to screen a library of 1.5 million compounds to identify compounds predicted to bind to a DNA binding pocket within the DNA binding domain of LIG1, thereby inhibiting DNA joining. Of the 192 candidates, ten compounds which inhibited purified LIG1 were also counter screened against LIG3, LIG4, in cell extract assays of DNA replication, base excision repair and non-homologous end joining. L82 was isolated as a specific uncompetitive inhibitor of LIG1 that stabilized complex formation between LIG1 and nicked DNA with an IC_50_ of 12 ± 2µM. L82 monotherapy was cytostatic and activated G1/S checkpoint in cancer cells 6.

We first tested cytotoxicity of L82 monotherapy in A2780 and A2780cis (Figure S6G). IC50 for cisplatin cytotoxicity was 38μM for A2780 and 24mΜ for A2780cis cells. In platinum resistant A2780cis cells, Cisplatin + L82 combination therapy significantly increased cytotoxicity (Figure S6H) implying that L82 is a platinum sensitizer. The data concurs with the platinum sensitization observed in LIG1 depleted ovarian cancer cells (Figure 2). We then proceeded to test if LIG1 blockade via L82 monotherapy could be a synthetic lethality strategy in DNA repair deficient cancer cells.

**Figure 4:**
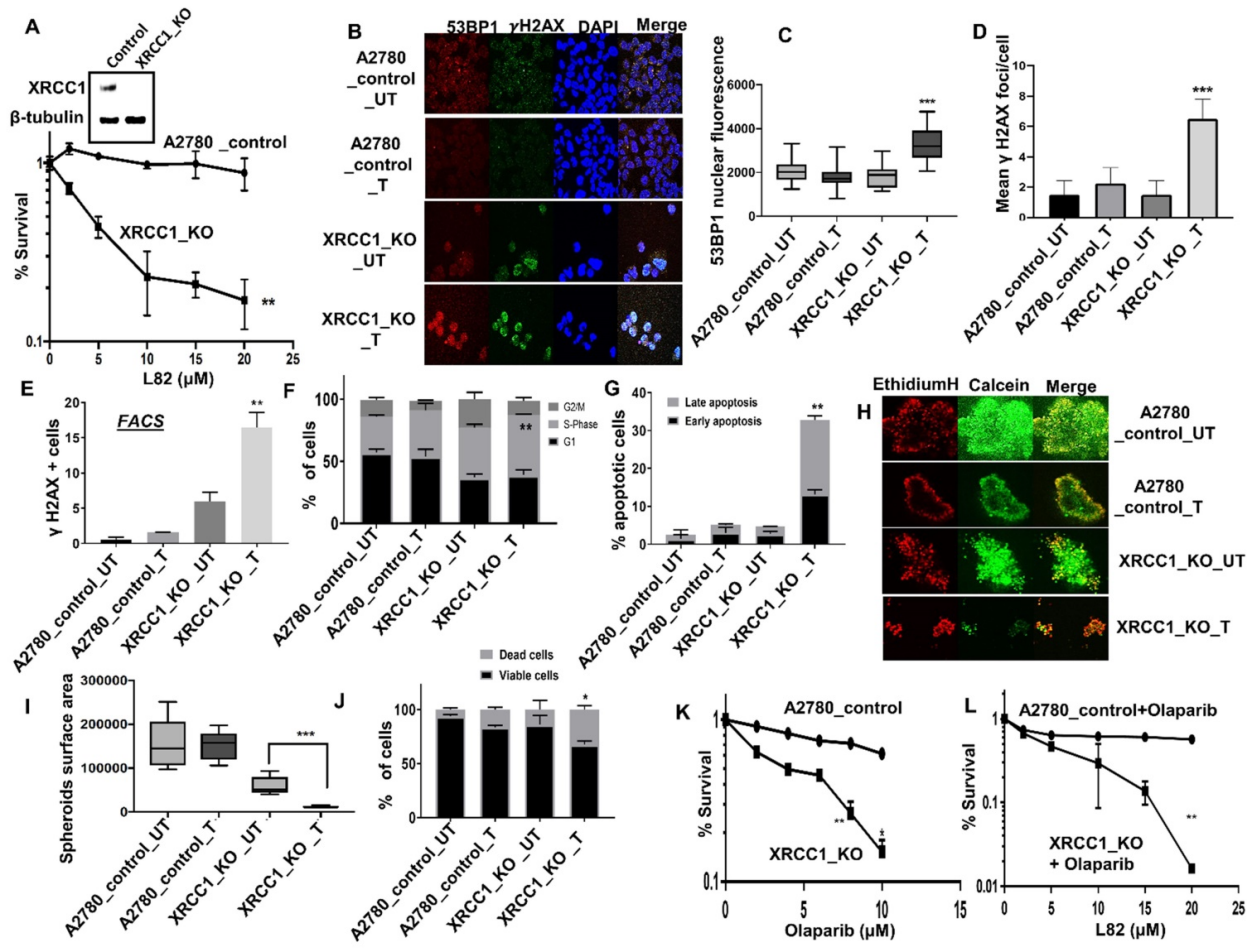
(**A**) Clonogenics survival assay for L82 sensitivity in A2780 control and A2780 (XRCC1_KO) (p-value was calculated as an average across control and KD cell line). (**B**) Representative photo micrographic images for immunofluorescence staining of γH2AX and 53BP1 in A2780 control and A2780 (XRCC1_KO) cells treated with L82 (10 μM) for 24 hrs. (**C**) Quantification of 53BP1 nuclear fluorescence by ImageJ software. (**D**) Quantification of γH2AX foci/cell by ImageJ software**.** Quantification of γH2AX positive cells by flow cytometry (**E**), Cell cycle analysis by flow cytometry (**F**) & Annexin V analysis by flow cytometry (**G**) in A2780 control and A2780 (XRCC1_KO) treated with L82 (10 μM) for 24 hrs. (**H**) Representative photomicrographic images of A2780 control and A2780 (XRCC1_KO) 3D-spheres treated with 10 μM of L82. (**I**) Quantification of spheroids size by ImageJ software. (**J**) quantification of spheroids cell viability by flow cytometry. (**K**) Olaparib sensitivity in A2780_Control and A2780_XRCC1_KO cells. (**L**) Cytotoxicity of Olaparib + L82 combination in A2780_Control and A2780_XRCC1_KO cells. Figures are representative of 3 or more experiments. Error bars represent standard error of mean between experiments. '*' = P-values < 0.05, '**' = P-values < 0.01, '***' = P-values < 0.001.

In platinum sensitive sporadic or BRCA germ-line deficient ovarian cancers, PARP inhibitor (Niraparib, Olaparib, Rucaparib) maintenance therapy has been shown to improve PFS in patients 1-3. We have previously shown that XRCC1, a key scaffolding protein and a partner for LIG3 *or LIG1* is a key predictor of platinum sensitivity in ovarian cancer 18. We have also recently generated XRCC1 knock out (KO) A2780 cells using CRISPR/Cas-9 methodology and demonstrated synthetic lethality with PARP inhibitors such as Olaparib and Talazoparib 19. To evaluate whether LIG1 inhibition is synthetically lethal in XRCC1 deficient cells, we tested L82 in a panel of cancer cell lines. As shown in Figure 4A**,** L82 was highly selectively toxic in XRCC1_KO_A2780 cells compared to control cells. Increased sensitivity to L82 in XRCC1_KO_A2780 cells resulted in increased in 53BP1 foci accumulation (Figure 4B-C), γH2AX foci accumulation (Figure 4B-E), S-phase cell cycle arrest (Figure 4F) and induction of apoptosis (Figure 4G). To recapitulate in an *in vivo* system, we then generated 3D-spheroids of XRCC1_KO_A2780 cells and control cells. Upon L82 treatment, in XRCC1-deficient A2780 spheroids, there was an accumulation of apoptotic cells (Figure 4H-J) as well as a reduction in spheroid size (Figure 4I) compared to XRCC1-proficient A2780 spheroids. For additional validation, we validated L82 activity in XRCC1 deficient and control HeLa cells (Figure S7A). As expected, L82 was selectively toxic in HeLa XRCC1-deficient cells compared to control HeLa cells (Figure S7B). Increased sensitivity was associated with DSB accumulation (Figure S7C), S-phase arrest (Figure S7D) and increased apoptotic cells (Figure S7E). L82 was also selectively toxic in XRCC1-deficient HeLa spheroids as evidenced by an accumulation of apoptotic cells as well as a reduction in spheroid size (Figure S7F-H) compared to XRCC1-proficient HeLa spheroids.

We then tested if Olaparib (PARP inhibitor) and L82 would be a viable combination therapy. As reported previously 19, Olaparib was selectively toxic in XRCC1_KO cells (Figure 4K). We observed significant selective toxicity when Olapraib (5µM) was combined with increasing doses of L82 (Figure 4L). Taken together, the data suggest that LIG1 specific inhibitor monotherapy or in combination with PARP inhibitor could be a novel synthetic lethality strategy in XRCC1 deficient ovarian cancers.

Previous studies suggest a redundancy of LIG3 and LIG1 during DNA replication and repair including BER. LIG3 has been shown to interact with XRCC1. Therefore, we first tested LIG3 levels in LIG1 depleted ovarian cancer cells. As shown in Figure S8A, we did not observe any increase in LIG3 protein level in LIG1 deficient cells. We then tested a previously isolated competitive small molecular inhibitor of both LIG1 and LIG3 (L67) 6. L67 has been shown to be cytotoxic either alone or in alkylating agent (MMS)^6^. Here we observed that L67 was selectively toxic in XRCC1 deficient cells at 5 µM and 10 µM doses compared to control cells (Figure S8B**)**.

**Figure 5:**
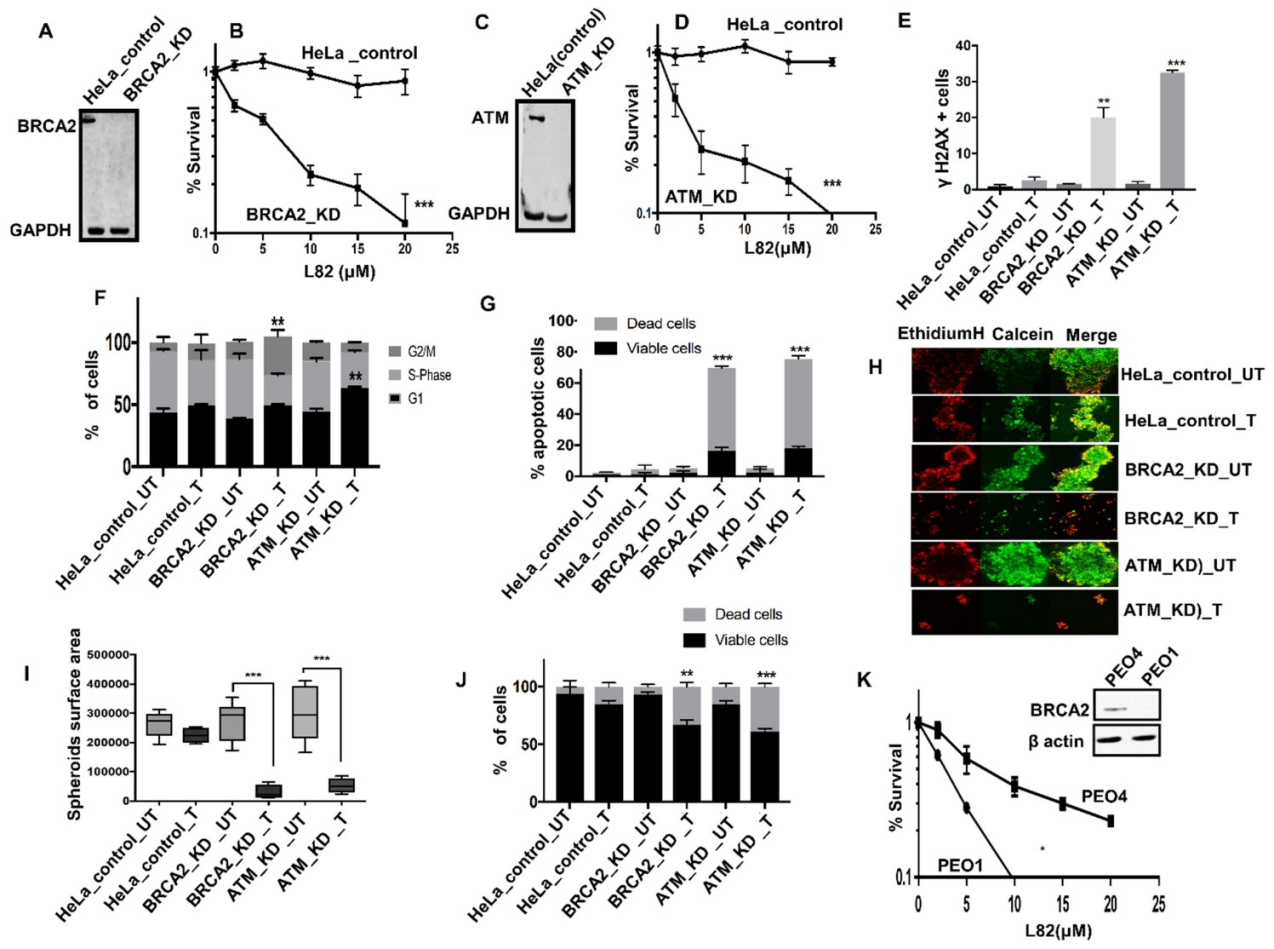
(**A**) western blot for BRCA2 knock down in HeLa SilenciX cells. (**B**) Clonogenics survival assay for L82 sensitivity in HeLa control and HeLa (BRCA2_KD) cells (p-value was calculated as an average across control and KD cell line). (**C**) western blot for ATM knockdown in HeLa SilenciX cells. (**D**) Clonogenics survival assay for L82 sensitivity in HeLa control and HeLa (ATM_KD) cells (p-value was calculated as an average across control and KD cell line). (**E**) Quantification of γH2AX positive cells by flow cytometry in HeLa control cells, HeLa (BRCA2_KD) and HeLa (ATM_KD) cells treated with L82 (10µM) for 24 h. (**F**) Cell cycle analysis by flow cytometry in HeLa control cells, HeLa (BRCA2_KD) and HeLa (ATM_KD) cells treated with L82 (10µM) for 24 h. (**G**) AnnexinV analysis for apoptotic cells in HeLa control cells, HeLa (BRCA2_KD) and HeLa (ATM_KD) cells treated with L82 (10µM) for 24 h. (**H**) Representative photomicrographic images of HeLa control, HeLa (BRCA2_KD) & HeLa (ATM_KD) 3D-spheres treated with 10 μM of L82. (**I**) Quantification of spheroids size by ImageJ software. (**J**) quantification of spheroids cell viability by flow cytometry. (**K**) L82 sensitivity in PEO1 and PEO4 cells. Figures are representative of 3 or more experiments. Error bars represent standard error of mean between experiments. '*' = P-values < 0.05, '**' = P-values < 0.01, '***' = P-values < 0.001.

**L82 is selectively toxic in BRCA2 or ATM deficient HeLa cells:** To evaluate if L82 is also selectively toxic in DSB repair deficient cancer cells, we tested in BRCA2 deficient HeLa (Figure 5A) and compared to controls. BRCA2 deficient cells were sensitive to L82 treatment compared to control (Figure 5B). Similarly, ATM deficient HeLa cells (Figure 5C-D) were sensitive to L82 treatment compared to control. Increased sensitivity in BRCA2 deficient cells were associated with DSB accumulation (Figure 5E), G2M cells cycle arrest (Figure 5F) and increased apoptosis (Figure 5G). Increased sensitivity in ATM deficient cells were associated with DSB accumulation (Figure 5E), G1 cells cycle arrest (Figure 5F) and increased apoptosis (Figure 5G). Upon L82 treatment, BRCA2-deficient or ATM deficient HeLa spheroids accumulated apoptotic cells along with reduced spheroid size (Figure 5H-J) compared to proficient HeLa spheroids. We then tested L82 cytotoxicity in PEO1 (BRCA2 deficient) and PEO4 (BRCA2 proficient) ovarian cancer cells. As shown in Figure 5K, L82 was selectively toxic in PEO1 cells compared to PEO4 cells.

## Discussion

The human ligases are essential for the maintenance of genomic integrity 8. LIG1 is involved in DNA replication, LP-BER, SSBR, NER and alt-NHEJ. LIG3 play important roles during SP-BER, SSBR, NER, alt-NHEJ, mitochondrial DNA replication and repair. LIG4 is a key player in NHEJ 8. This is the first comprehensive study of LIG1, LIG3 and LIG4 in epithelial ovarian cancers.

In multivariate analyses LIG1 protein was identified as independent marker of poor clinical outcome. Polymorphic variants of LIG1 may influence lung cancer, upper GI cancers 20 and head & neck cancers 21. Platinum induced intra-strand cross links and oxidative DNA base lesions are repaired through NER and LP-BER respectively. In previous studies, increased levels of LIG1 have been observed in cancer cells lines compared with normal cells, likely related to increased proliferation 6. We observed that cisplatin treatment increased LIG1 protein levels in ovarian cancer cells. When we depleted LIG1, we observed increased platinum sensitivity implying that LIG1 is predictor of platinum sensitivity.

For LIG3 we observed both cytoplasmic and nuclear staining. However, only cytoplasmic overexpression of LIG3 was linked with poor outcomes in patients. In addition, when cytoplasmic and nuclear expression was combined, only tumours with high cytoplasmic/low nuclear LIG3 co-expression had worse PFS and OS compared to tumours with low cytoplasmic/low nuclear LIG3 co-expression. As *LIG3* gene encodes three distinct DNA ligase polypeptides (including nuclear LIG3α, mitochondrial LIG3α and a germ cell-specific LIG3β) 8, 22, we speculate that the cytoplasmic expression observed here may represent the mitochondrial form of LIG3 12, 13. Platinum induced mitochondrial DNA damage can promote cellular cytotoxicity 23. Altered mitochondrial DNA repair 24 as well as replication capacity 25 may influence response to platinum therapy and increased mitochondrial LIG3 could contribute to platinum resistance 12, 13. However, mechanistic studies will be required to confirm the role of mitochondrial LIG3 in platinum resistance in ovarian cancers.

In the current study low LIG4 expression predicted resistance to platinum therapy and poor survival. In nasopharyneal carcinomas, low LIG4 level was associated with worse survival 26. Reduced LIG4 level has been described in cancer cell lines 6. LIG4 deficiency contributes to abnormal DSB repair in chronic myeloid leukemia cells 27. Germ-line mutation and inactivation of LIG4 has been associated with cancer predisposition and clinical immunodeficiency syndromes 14, 28. *LIG4* variants may result in dysfunctional NHEJ and single nucleotide polymorphisms in *LIG4* may be associated with an increased risk of developing ovarian cancer 29. *LIG4* genetic polymorphisms have also been linked with breast cancer 30 and myeloma risk 31. Our data suggests that LIG4 deficiency may promote a 'mutator phenotype' leading on to aggressive cancers. In a recent study McCormick et al demonstrated defects in NHEJ (which is independent of HR function) in about 40% of ovarian cancer cells which may be a predictor of resistance to PARP inhibitor (PARPi) therapy 32. In contrast, homologous recombination deficiency (HRD) due to genetic or epigenetic alterations in HR pathway genes has been observed in up to 50% of epithelial ovarian cancers and is related to sensitivity to platinum and PARPi therapy. 53BP1 deficiency can activate HR which in turn result in resistance to PARPi and platinating agents 33. It is likely that LIG4 deficiency observed in the current study may reflect NHEJ defective tumours. As NHEJ and HR compete for DSB repair, we speculate that LIG4 loss will increase HR resulting in resistance to platinum chemotherapy. Detailed functional studies will be required to confirm this hypothesis.

Human ligases have emerged as promising targets for cancer therapy 34. L82 is a specific small molecule inhibitor of LIG1 6. L82 monotherapy in MCF7 breast cancer cells was shown to be cytostatic with activation G1/S checkpoint 6. Here we show that LIG1 blockade could be an attractive synthetic lethality strategy in XRCC1 deficient cancer cells. We speculate a synthetic lethality relationship exists between XRCC1 and LIG1 for the following reasons; 1) XRCC1 is a key player in BER and SSBR. 2) XRCC1 also has well recognised roles during processing of replication forks and replication stress. 3) XRCC1 deficiency will lead to replication stress and accumulation of SSBs. 4) LIG1 blockade induced replication stress is amplified in XRCC1 deficient cells because of the role of LIG3 as a back-up to LIG1 in DNA replication leading onto accumulation of SSB which get converted to DSB during replication. 5) Excessive DSB promote apoptotic cell death. However, the detailed molecular mechanisms of the role of LIG1 in ovarian cancer DNA repair are an area of ongoing investigation and as such are a limitation of the current study. We have recently shown that PARP inhibitors (Olaparib, Talazoparib) induce selective toxicity in XRCC1 deficient ovarian and breast cancer cells. A recent study by Pillay *et al*. in ovarian cancer cells has also revealed that the response to DNA repair inhibitors such as those targeting PARP or PARG may be dependent on DNA replication vulnerabilities in ovarian cancer cells 35. Taken together, our data would suggest that XRCC1 deficient tumors are selectively sensitive to DNA repair inhibitors such as those targeting PARP or LIG1.

LIG1 blockade could also be a promising alternative synthetic lethality strategy in BRCA2 deficient cells. BRCA2, besides its critical role in HRR 36, also protects stalled replication forks through its ability to stabilize RAD51 filaments 37. We speculated that in BRCA2 deficient cells that accumulate replication fork intermediates, LIG1 blockade would result in the accumulation of toxic DNA intermediates which get converted to DSBs leading to apoptotic cell death. Moreover, LIG1 blockade will impair LP-BER and replication resulting in accumulation of SSBs, which if unrepaired, result in DSB generation. In cells deficient in homologous recombination repair (HRR), DSBs would persist and lead to synthetic lethality. Accordingly, in BRCA2 deficient we observed selective toxicity associated with accumulation of DSBs, G2/M cell cycle arrest and increased apoptosis. ATM is a key damage signalling protein and critical for DSB repair 38. ATM deficient cells were also sensitive to LIG1 blocakde which was linked with DSB accumulation, G1 cell cycle arrest and apoptosis. A limitation to the study is that we have only tested synthetic lethality in BRCA2 or ATM deficient HeLa cancer cell line models. Further studies in BRCA2 or ATM deficient ovarian cancer or breast cancer models will be required to confirm our preliminary observations. In the current study we have tested the potential of LIG1 inhibitors in cell line models only. Additional studies in organoids as well as in vivo xenograft studies including in patient derived models will be required to validate our observations. Although we tested only one available small molecular inhibitor of LIG1, additional more potent LIG1 inhibitors will also need to be isolated and evaluated to further validate our observations.

In conclusion, the 'proof of concept' study presented here suggests that LIG1 blockade is an attractive strategy and pharmaceutical development of LIG1 inhibitors is required to accelerate clinical application.

## Supplementary Material

Supplementary materials and methods, figures and tables.

## Figures and Tables

**Table 1:** LIG1 nuclear protein expression and epithelial ovarian cancers.

Parameter	Low nuclear LIG1 N(%)	High nuclear LIG1 N(%)	P-Value
***Pathological Type***			***<0.0001***
Serous	80 (32.5)	166 (67.5)	
Mucinous	37 (71.2)	15 (28.8)	
Endometrioid	49 (70.0)	21 (30.0)	
Clear cell carcinoma	23 (65.7)	12 (34.3)	
Mixed	8 (47.1)	9 (52.9)	
Others	7 (46.7)	8 (53.3)	
***FIGO Stage***			***<0.0001***
I	96 (61.5)	60 (38.5)	
II	33 (48.5)	35 (51.5)	
III	62 (35.4)	113 (64.6)	
IV	7 (29.2)	17 (70.8)	
***Tumour Grade***			***<0.0001***
G1	34 (63.0)	20 (37.0)	
G2	45 (61.6)	28 (38.4)	
G3	99 (38.7)	157 (61.3)	
***Surgical Optimal Debulking***			***0.004***
Optimally Debulked	162 (50.9)	156 (49.1)	
Not Optimally Debulked	31 (34.1)	60 (65.9)	
***Residual Tumour***			***0.014***
None/Microscopic/<1cm	153 (50.7)	149 (49.3)	
1-2 cm / >2cm	39 (36.8)	67 (63.2)	
***Measurable Disease Before Chemotherapy***			***0.011***
Non- measurable	140 (52.0)	129 (48.0)	
Measurable	50 (38.5)	80 (61.5)	
***Platinum sensitivity***			0.063
Sensitive	155 (48.9)	162 (51.1)	
Resistant	24 (36.4)	42 (63.6)	

## Abbreviations

ATM: ATM Serine/Threonine Kinase
BRCA2: Breast And Ovarian Cancer Susceptibility Protein 2
DNA: Deoxyribose nucleic acid
DSB: Double Strand Break
FACS: Fluorescence cell sorting
FIGO: International Federation of Obstetricians and Gynaecologists
HR: Homologous recombination
LIG: Ligase
mRNA: Messenger Ribose nucleic acid
NER: Nucleotide excision repair
NHEJ: Non-homologous end joining
OS: Overall Survival
PFS: Progression Free Survival
PARP: Poly(ADP-Ribose) Polymerase
SP-BER: Short-patch base excision repair
SSBR: Single strand break repair
XRCC1: X-Ray Repair Cross Complementing 1

## References

[1] Konstantinopoulos PA, Lheureux S, Moore KN (2020). PARP Inhibitors for Ovarian Cancer: Current Indications, Future Combinations, and Novel Assets in Development to Target DNA Damage Repair. Am Soc Clin Oncol Educ Book.

[2] Lord CJ, Ashworth A (2017). PARP inhibitors: Synthetic lethality in the clinic. Science.

[3] Lord CJ, Tutt AN, Ashworth A (2015). Synthetic lethality and cancer therapy: lessons learned from the development of PARP inhibitors. Annu Rev Med.

[4] D'Andrea AD (2018). Mechanisms of PARP inhibitor sensitivity and resistance. DNA Repair (Amst).

[5] Francica P, Rottenberg S (2018). Mechanisms of PARP inhibitor resistance in cancer and insights into the DNA damage response. Genome Med.

[6] Chen X, Zhong S, Zhu X, Dziegielewska B, Ellenberger T, Wilson GM (2008). Rational design of human DNA ligase inhibitors that target cellular DNA replication and repair. Cancer Res.

[7] Tomkinson AE, Vijayakumar S, Pascal JM, Ellenberger T (2006). DNA ligases: structure, reaction mechanism, and function. Chem Rev.

[8] Ellenberger T, Tomkinson AE (2008). Eukaryotic DNA ligases: structural and functional insights. Annu Rev Biochem.

[9] Tomkinson AE, Levin DS (1997). Mammalian DNA ligases. Bioessays.

[10] Arakawa H, Iliakis G (2015). Alternative Okazaki Fragment Ligation Pathway by DNA Ligase III. Genes (Basel).

[11] Cappelli E, Taylor R, Cevasco M, Abbondandolo A, Caldecott K, Frosina G (1997). Involvement of XRCC1 and DNA ligase III gene products in DNA base excision repair. J Biol Chem.

[12] Gao Y, Katyal S, Lee Y, Zhao J, Rehg JE, Russell HR (2011). DNA ligase III is critical for mtDNA integrity but not Xrcc1-mediated nuclear DNA repair. Nature.

[13] Lakshmipathy U, Campbell C (2000). Mitochondrial DNA ligase III function is independent of Xrcc1. Nucleic Acids Res.

[14] Jiang J, Tang W, An Y, Tang M, Wu J, Qin T (2016). Molecular and immunological characterization of DNA ligase IV deficiency. Clin Immunol.

[15] Frank KM, Sekiguchi JM, Seidl KJ, Swat W, Rathbun GA, Cheng HL (1998). Late embryonic lethality and impaired V(D)J recombination in mice lacking DNA ligase IV. Nature.

[16] Gyorffy B, Lanczky A, Szallasi Z (2012). Implementing an online tool for genome-wide validation of survival-associated biomarkers in ovarian-cancer using microarray data from 1287 patients. Endocr Relat Cancer.

[17] Mesquita KA, Ali R, Doherty R, Toss MS, Miligy I, Alblihy A (2021). FEN1 Blockade for Platinum Chemo-Sensitization and Synthetic Lethality in Epithelial Ovarian Cancers. Cancers (Basel).

[18] Abdel-Fatah T, Sultana R, Abbotts R, Hawkes C, Seedhouse C, Chan S (2013). Clinicopathological and functional significance of XRCC1 expression in ovarian cancer. Int J Cancer.

[19] Ali R, Alabdullah M, Alblihy A, Miligy I, Mesquita KA, Chan SY (2020). PARP1 blockade is synthetically lethal in XRCC1 deficient sporadic epithelial ovarian cancers. Cancer Lett.

[20] Lee YC, Morgenstern H, Greenland S, Tashkin DP, Papp J, Sinsheimer J (2008). A case-control study of the association of the polymorphisms and haplotypes of DNA ligase I with lung and upper-aerodigestive-tract cancers. Int J Cancer.

[21] Michiels S, Danoy P, Dessen P, Bera A, Boulet T, Bouchardy C (2007). Polymorphism discovery in 62 DNA repair genes and haplotype associations with risks for lung and head and neck cancers. Carcinogenesis.

[22] Simsek D, Jasin M (2011). DNA ligase III: a spotty presence in eukaryotes, but an essential function where tested. Cell Cycle.

[23] Wisnovsky SP, Wilson JJ, Radford RJ, Pereira MP, Chan MR, Laposa RR (2013). Targeting mitochondrial DNA with a platinum-based anticancer agent. Chem Biol.

[24] Zinovkina LA (2018). Mechanisms of Mitochondrial DNA Repair in Mammals. Biochemistry (Mosc).

[25] Ruhanen H, Ushakov K, Yasukawa T (2011). Involvement of DNA ligase III and ribonuclease H1 in mitochondrial DNA replication in cultured human cells. Biochim Biophys Acta.

[26] Kim DH, Oh SY, Kim SY, Lee S, Koh MS, Lee JH (2014). DNA ligase4 as a prognostic marker in nasopharyngeal cancer patients treated with radiotherapy. Asian Pac J Cancer Prev.

[27] Sallmyr A, Tomkinson AE, Rassool FV (2008). Up-regulation of WRN and DNA ligase IIIalpha in chronic myeloid leukemia: consequences for the repair of DNA double-strand breaks. Blood.

[28] Chistiakov DA, Voronova NV, Chistiakov AP (2009). Ligase IV syndrome. Eur J Med Genet.

[29] Assis J, Pereira D, Medeiros R (2013). Ovarian cancer and DNA repair: DNA ligase IV as a potential key. World J Clin Oncol.

[30] Kuschel B, Auranen A, McBride S, Novik KL, Antoniou A, Lipscombe JM (2002). Variants in DNA double-strand break repair genes and breast cancer susceptibility. Hum Mol Genet.

[31] Roddam PL, Rollinson S, O'Driscoll M, Jeggo PA, Jack A, Morgan GJ (2002). Genetic variants of NHEJ DNA ligase IV can affect the risk of developing multiple myeloma, a tumour characterised by aberrant class switch recombination. J Med Genet.

[32] McCormick A, Donoghue P, Dixon M, O'Sullivan R, O'Donnell RL, Murray J (2017). Ovarian Cancers Harbor Defects in Nonhomologous End Joining Resulting in Resistance to Rucaparib. Clin Cancer Res.

[33] Mirman Z, de Lange T (2020). 53BP1: a DSB escort. Genes Dev.

[34] Tomkinson AE, Howes TR, Wiest NE (2013). DNA ligases as therapeutic targets. Transl Cancer Res.

[35] Pillay N, Tighe A, Nelson L, Littler S, Coulson-Gilmer C, Bah N (2019). DNA Replication Vulnerabilities Render Ovarian Cancer Cells Sensitive to Poly(ADP-Ribose) Glycohydrolase Inhibitors. Cancer Cell.

[36] Chen CC, Feng W, Lim PX, Kass EM, Jasin M (2018). Homology-Directed Repair and the Role of BRCA1, BRCA2, and Related Proteins in Genome Integrity and Cancer. Annu Rev Cancer Biol.

[37] Feng W, Jasin M (2017). Homologous Recombination and Replication Fork Protection: BRCA2 and More!. Cold Spring Harb Symp Quant Biol.

[38] Shiloh Y, Ziv Y (2013). The ATM protein kinase: regulating the cellular response to genotoxic stress, and more. Nat Rev Mol Cell Biol.

