# Supplementary material for: Ligase 1 is a predictor of platinum resistance and its blockade is synthetically lethal in XRCC1 deficient epithelial ovarian cancers
Source: Theranostics. 2021 Jul 25;11(17):8350–61. doi: 10.7150/thno.51456 (PMC8344016; doi:10.7150/thno.51456)
Supplement: Supplementary file 1 — Supplementary materials and methods, figures and tables. [file thnov11p8350s1.pdf]

SUPPLEMENTARY MATERIAL

METHODS

Patient cohort: A total of 525 patients with histologically confirmed ovarian cancer and treated from 1997 to 2010 at Nottingham University Hospitals (NUH) were included in this study. They were staged as per the International Federation of Obstetricians and Gynaecologists (FIGO) Staging System for Ovarian Cancer. Clinical follow-up information was obtained by review of the patients' records and retrospectively analysed. Overall Survival was calculated from the operation date until the 1st of October 2016 when any remaining survivors were censored. Progression-free survival was calculated from the date of the initial surgery to disease progression or from the date of the initial surgery to the last date known to be progression-free for those censored. Patients' demographics are summarized in Table S1. Platinum resistance was defined as patients who had progression during first-line platinum chemotherapy or relapse within 6 months after completion of platinum treatment.

Tissue microarray (TMA) and immunohistochemistry (IHC): TMAs were constructed. Briefly, triplicate tissue cores with a diameter of 0.6mm were taken from the tumour and arrayed into a recipient paraffin block using a tissue puncher/arrayer (Beecher Instruments, Silver Spring, MD, USA). Four micron sections of the tissue array block were cut and placed on Surgipath X-tra Adhesive microscope slides (Leica Microsystems) for immunohistochemical staining. Immunohistochemical staining was performed using Novocastra Novolink polymer detection system according to manufacturer instructions (Leica Microsystems, Newcastle, UK). Pre-treatment of TMA sections was performed with citrate buffer (pH 6.0, 20 min, Microwave). Then were incubated for 60 min at room temperature with 1:25 anti-LIG1 rabbit monoclonal antibody (ab177946, ABCAM). For LIG3, TMA sections were overnight incubated at room temperature with 1:100 of anti-LIG3 rabbit polyclonal antibody (HPA006723, SIGMA). TMA sections were stained with anti LIG4 antibody

(HPAA001334, SIGMA) at concentration of (1:100) for 60 min incubation in room temperature. Sections were counterstained with haematoxylin. Negative controls with no primary antibody were included in each run. Cases with multiple cores were scored and the average was used as the final score.

Evaluation of immune staining: The tumour cores were evaluated by MA (joint first author) and an expert pathologist in two different settings. Both were blinded to the clinico-pathological characteristics of patients. There was excellent intra and inter-observer agreements (k > 0.9; Cohen's κ and multi-rater κ tests, respectively). Whole field inspection of the core was scored, the sub cellular localisation of each marker was identified (nuclear, cytoplasm, cell membrane), and the optimal scoring methodology was applied in each case. Intensities of subcellular compartments were each assessed and grouped as follows: 0 = no staining, 1 = weak staining, 2 = moderate staining, 3 = strong staining. The percentage of tumour cells in each category was estimated (0–100%). H-score (range 0–300) was calculated by multiplying the intensity of staining and the percentage of staining. The median was utilised as the optimal cut-off for all the tested antibodies. For LIG1, low/negative nuclear expression was defined by H-score of < 60. For LIG3, low/negative nuclear or cytoplasmic expression was defined the H-score of ≤ 0 and ≤ 100 respectively. For LIG4, a H score of ≤190 nuclear was considered as low/negative. Not all cores within the TMA were suitable for IHC analysis due to missing cores or absence of tumour cells.

Statistical analysis: This was performed using SPSS v 22 (Chicago, IL, USA) for Windows. Association with clinical and pathological parameters using categorised data was examined using Chi-squared test. All tests were 2-tailed. Survival rates were determined using Kaplan–Meier method and compared by the log-rank test. All analyses were conducted using Statistical Package for the Social Sciences (SPSS, version 22, Chicago, IL, USA) software for windows. P value of less than 0.05 was identified as statistically significant. This study was carried out

in accordance with the declaration of The Helsinki and ethical approval which was obtained from the Nottingham Research Ethics Committee (REC Approval Number 06/Q240/153).

***LIG1, LIG3 and LIG4* mRNA expression and human epithelial ovarian cancers:** Predictive and prognostic significance of *LIG1, LIG3 and LIG4* mRNA expression mRNA expression was investigated in publicly available ovarian tumour gene expression data sets (http://kmplot.com/analysis/index.php?p=service&cancer=ovar) [1]. A total of 1075 Serous cystadenocarcinomas were included in this analysis.

Pre-clinical study

Compounds and antibodies: LIG1, ATM, BRCA2, pATM and pCHK1 antibodies were purchased from Abcam, UK. XRCC1 antibody was purchased from Thermofisher scientific. Histone H2AX phosphorylated at Ser^{139} (γH2AX) were purchased from Millipore, UK. Calcein AM and Ethidium homodimer -1 were purchased from ThermoFisher, UK. Cisplatin solution (1 mg/ml) was obtained from the Department of Pharmacy, Nottingham University Hospitals, Nottingham,UK. A specific small molecule inhibitor of LIG1 (L82) was isolated as described previously [2]. L82 was purchased from Chemdiv (San Diego, CA, USA). 10 mM stocks were prepared in DMSO and stored at −20° C. The molecular mass and purity of L82 was confirmed by mass spectrometry in the University of Maryland School of Pharmacy facility. Is this true-what about the other inhibitors?

Cell lines and culture: A2780, A2780cis, PE01 and PE04 ovarian cancer cell lines were purchased from American Type Culture Collection (ATCC, Manassas, USA). Cells were cultured in RPMI medium supplemented with 10% FBS and 1% penicillin streptomycin. Cisplatin resistance in A2780cis were maintained by adding 1μM Cisplatin to the culture medium every 2-3 passages. BRCA2-deficient HeLa SilenciX cells and controls BRCA2-proficient HeLa SilenciX cells, XRCC1-deficient HeLa SilenciX cells and controls XRCC1-proficient HeLa SilenciX cells, ATM-deficient HeLa SilenciX cells and controls ATM-

proficient HeLa SilenciX cells were all purchased from Tebu-Bio (www.tebu-bio.com). SilenciX cells were grown in Dulbecco's Modified Eagle's Medium supplemented with 10% FBS, 1% penicillin/streptomycin, and 125 µg/mL hygromycin B. cell lines were tested for mycoplasma routinely every 3 months using mycoProbe mycoplasma detection kit (R&D systems). All cell lines were used between 15 passages window.

Generation of XRCC1 knock out using CRISPR-cas9: A2780 cells were transfected with oligonucleotides carrying gRNA silencing XRCC1 cloned in Plv-U6g-EPCG plasmid (Sigma, UK). Briefly, cells were seeded at 50-60% confluency in 6-well plates overnight. Cells were transfected with 2-3 µg of DNA using Lipofectamine 3000 (Invitrogen, UK) in Opti-MEM medium. Puromycin (10 µg/ml) was used for the selection of desired clones for 14 days.

LIG1 knock down using siRNAs: The siRNA constructs for LIG1 were obtained from Invitrogen, UK siRNA 1 (cat. no: 4390824 Assay ID: S8173) second siRNA construct for LIG1 (cat. no.4390824, Assay ID: S8174) .. 1x10 ^{6}Cells were seeded in T25 cell culture flasks overnight. SiRNA constructs were transfected using Lipofecamine 3000 reagent (Invitrogen, UK) as per the manufacturer's protocol in Opti-MEM low serum medium. Transfection efficiency was confirmed at day 3 and day 5 using western blotting.

Western blotting: Cells were lysed in RIPA buffer (Sigma Aldrich) complemented with protease inhibitor (Sigma) and phosphatase inhibitor cocktail 1 and 2 (Sigma). Protein quantification was performed using BCA colorimetric kit (Thermofisher, UK). Samples were run on SDS-PAGE gel (4-12%) bis-tris. Membranes were incubated with anti LIG1 (4°C/overnight) and anti-GADPH (1 hour room temperature). Membranes were then labelled with infrared dye labeled secondary antibodies (Li-cor) [IRDye 800CW Donkey Anti-Rabbit IgG (H+L) and IRDye 680CW Donkey Anti-Mouse IgG (H+L)] for 1 hour at room temperature. Protein detection and quantification was determined by scanning the membranes on Licor-Odyssey's Scanner (Licor,Biosciences) at the predefined intensity fluorescence.

Clonogenic survival assays: For ovarian cancer cell lines 200 cells /well were seeded in 6-well plates. For HeLa cell lines 250 cells/well were seeded in 6- well plates overnight then treated with the indicated doses of Cisplatin or DNA Ligases inhibitors. Platting efficiency for each cell line was determined prior to the drug sensitivity experiments. The plates were left in the incubator for 14 days, after incubation colonies were washed with PBS and stained with crystal violet, acetic acid and methanol mixture and counted. Survival fraction (SF) were calculated using the formula SF = no. of colonies formed after treatment/no. of cells seeded x platting efficiency. Number of colonies counted were normalised relative to the count of untreated wells which were considered as 100% survival.

Cell cycle and apoptosis by Flow Cytometry: $1x10^5$ Cells per well were seeded in 6-well plates overnight. Cells were treated with Cisplatin (1µM) for A2780 cells and (5µM) for A2780 cis cells. After 24 hours Cells were trypsinized and washed with ice cold PBS, then fixed in 70% ethanol for at least 30 mins. After removal of the fixative solution by centrifugation cells were stained with phospho Histone (γH2AX) Ser139. Cells were then treated with RNase and DNA content were stained with 10ug/ml propidium iodide (Sigma Aldrich) in PBS. For Apoptosis detection, cells were collected by trypsinization after 24 hours washed and analysed using annexinV detection kit (BD biosciences). Samples were analysed on FC500 flow cytometer (Beckman Coulter) and data were analysed using Weasel software. Data were generated using GraphPad Prism7 software.

Confocal microscopy: Cells were seeded on the cover slips overnight, then treated with DNA LIG1 inhibitor (10µM) for 24 hrs. Cells were fixed with 4% (w/v) paraformaldehyde for 30 min, permeabilized with 0.1% (w/v) Triton X100 (ThermoFisher) for 30 min and blocked with 3% (w/v) BSA for 1 h. Cells were incubated with anti- 53BP1 (Cell Signalling, catalogue no. 4937S) and anti γH2AX (Merck millipore clone JBW301) overnight at 4°C. Slides were prepared in duplicates. Imaging was carried out using Leica SP2 confocal laser scanning

microscope. For analysis a minimum of 100 cells per slide were counted. Nuclear fluorescence was quantified for γH2AX and 53BP1 using ImageJ software. Values were plotted in GraphPad Prism 7.

Generation of 3D spheroids: Cells ($4x10^4$) were seeded in ultra-low attachment 6-well plates using promo cell tumour spheres medium. After that cells were left to form spheres for 3-weeks. Spheroids were treated with 10 µM of DNA LIG1 inhibitor for 48 hrs. Then, spheres were fixed with formaldehyde (3.7%, w/v) and stained with 2 µM calcein AM and 1.5 µM ethidiumhomodimer-1. Imaging was carried out using Leica SP2 confocal laser scanning microscope. Images were analyzed by ImageJ software.

Targeted next generation sequencing and bioinformatics: Genomic DNA was extracted from cell lines using the PicoPure™ DNA Extraction Kit (Thermofisher,UK). Targeted next generation sequencing was used to identify genomic variants in platinum sensitive (A2780) and platinum resistant derivatives (A2780cis). The SureSelect All Exon V5 kit (Agilent Technologies) was used to enrich for protein coding regions and sequencing performed using an Illumina NextSeq500 sequencer with paired end reads (150bp) and a minimum of 88million reads generated per sample. Raw reads were fastq formatted. Contaminating adapter sequences and low-quality sequences were processed using Skewer [3]. Quality processed reads were aligned to the HG19 reference genome using BWA [4], duplicate alignments identified and processed using PicardTools, and realignment completed using the Abra assembly based realigner [5] to enhance detection of insertion/deletion variants. Variant calling and filtering was completed using Samtools/Bcftools (v1.3.1) [6]. Variants, in variant call format (VCF), associated with Platinum resistance were identified using vcf tools [7]. Variants were annotated and functional significance assessed using the Ensembl Variant Effect Predictor tool [8]. Library preparation and sequencing was conducted by Source Biosciences (Nottingham, UK). Genes affecting the LIG1 mammalian interactome were identified using

the BioGrid and KEGG database[9][10]. The gene list from the *LIG1* interactome identified in its associated KEGG pathways was interrogated in the Pt sensitive A2780 and PE01 and A2780cis and PE04 Pt resistant cell lines. Exome sequencing dataset identified gene variants. The biological function of each gene variant was identified using Webgestalt gene set analysis tool [11] and ORA (overrepresentation enrichment analysis based on gene ontology (GO)-annotated biological processes) [12]. The ratio of observed versus expected number of genes in the category was recorded for each significant category using the enrichment ratio (R) scores. The full NGS data is available at https://www.ncbi.nlm.nih.gov/sra/PRJNA731652.

Supplementary References

[1] Gyorffy B, Lanczky A, Szallasi Z. Implementing an online tool for genome-wide validation of survival-associated biomarkers in ovarian-cancer using microarray data from 1287 patients. Endocr Relat Cancer. 2012;19:197-208.
[2] Chen X, Zhong S, Zhu X, Dziegielewska B, Ellenberger T, Wilson GM, et al. Rational design of human DNA ligase inhibitors that target cellular DNA replication and repair. Cancer Res. 2008;68:3169-77.
[3] Jiang H, Lei R, Ding SW, Zhu S. Skewer: a fast and accurate adapter trimmer for next-generation sequencing paired-end reads. BMC bioinformatics. 2014;15:182.
[4] Li H, Durbin R. Fast and accurate long-read alignment with Burrows-Wheeler transform. Bioinformatics. 2010;26:589-95.
[5] Mose LE, Wilkerson MD, Hayes DN, Perou CM, Parker JS. ABRA: improved coding indel detection via assembly-based realignment. Bioinformatics. 2014;30:2813-5.
[6] Li H, Handsaker B, Wysoker A, Fennell T, Ruan J, Homer N, et al. The Sequence Alignment/Map format and SAMtools. Bioinformatics. 2009;25:2078-9.
[7] Danecek P, Auton A, Abecasis G, Albers CA, Banks E, DePristo MA, et al. The variant call format and VCFtools. Bioinformatics. 2011;27:2156-8.
[8] McLaren W, Gil L, Hunt SE, Riat HS, Ritchie GR, Thormann A, et al. The Ensembl Variant Effect Predictor. Genome Biol. 2016;17:122.
[9] Kanehisa M, Goto S. KEGG: kyoto encyclopedia of genes and genomes. Nucleic acids research. 2000;28:27-30.
[10] Oughtred R, Stark C, Breitkreutz BJ, Rust J, Boucher L, Chang C, et al. The BioGRID interaction database: 2019 update. Nucleic acids research. 2019;47:D529-D41.
[11] Wang J, Vasaikar S, Shi Z, Greer M, Zhang B. WebGestalt 2017: a more comprehensive, powerful, flexible and interactive gene set enrichment analysis toolkit. Nucleic Acids Res. 2017;45:W130-W7.
[12] The Gene Ontology C. Expansion of the Gene Ontology knowledgebase and resources. Nucleic Acids Res. 2017;45:D331-D8.

Table S1: Patient demographics

Characteristics	Number	Percentages
Pathology		
Serous cystadenocarcinoma	290	55.2%
Endometrioid	82	15.6%
Clear cell carcinoma	48	9.1%
Mucinous cystadenocarcinoma	60	11.4%
Others	18	3.4%
Mixed	17	3.2%
Grade		
1	68	12.9%
2	87	16.5%
3	306	58.2%
Residual tumour		
None/Microscopic	265	50.4%
<1cm	82	15.6%
>1-2 cm	29	5.5%
>2cm	110	20.9%
FIGO Stage		
I	182	34.6%
II	76	14.4%
III	213	40.5%
IV	32	6%
Chemotherapy		
Carboplatin monotherapy	163	31%
Carboplatin + Paclitaxel	177	33.7%
Platinum sensitivity		
Sensitive	376	71.6%
Resistant	85	16.1%
Unknown	62	11.8%
Relapse status		
Progression-free	238	45.3%
Progressed/relapsed	243	46.2%
Unknown	44	8.3%
Survival status		
Living	199	37.9%
Dead	286	54.4%

Unknown	40	7.6%

Table S2: LIG3 cytoplasmic protein expression and epithelial ovarian cancers.

**Parameter**	**Low cyto LIG3 N (%)**	**High cyto LIG3 N(%)**	**P-Value**
Pathological Type			0.458
Serous	114 (48.5)	121 (51.5)	
Mucinous	30 (61.2)	19 (38.8)	
Endometrioid	36 (57.1)	27 (42.9)	
Clear cell carcinoma	20 (60.6)	13 (39.4)	
Mixed	10 (58.8)	7 (41.2)	
Others	7 (53.8)	6 (46.2)	
FIGO Stage			***0.002***
I	94 (64.8)	51 (35.2)	
II	26 (41.3)	37 (58.7)	
III	84 (49.7)	85 (50.3)	
IV	9 (37.5)	15 (62.5)	
Tumour Grade			***0.028***
G1	35 (67.3)	17 (32.7)	
G2	29 (43.3)	38 (56.7)	
G3	124 (50.4)	122 (49.6)	
Surgical Optimal Debulking			0.162
Optimally Debulked	168 (55.6)	134 (44.4)	
Not Optimally Debulked	40 (47.1)	45 (52.9)	
Residual Tumour			***0.001***
None/Microscopic/<1cm	168 (58.9)	117 (41.1)	
1-2 cm / >2cm	40 (39.6)	61 (60.4)	
Measurable Disease Before Chemotherapy			***0.006***
Non- measurable	149 (58.7)	105 (41.3)	
Measurable	53 (43.4)	69 (56.6)	
Platinum sensitivity			***0.025***
Sensitive	163 (54.2)	138 (45.8)	
Resistant	23 (38.3)	37 (61.7)	

Table S3: LIG3 nuclear protein expression and epithelial ovarian cancers.

Parameter	Low nuc LIG3 N (%)	High nuc LIG3 N (%)	P-Value
Pathological Type			***0.017***
Serous	113 (48.1)	122 (51.9)	
Mucinous	34 (69.4)	15 (30.6)	
Endometrioid	38 (60.3)	25 (39.7)	
Clear cell carcinoma	19 (57.6)	14 (42.4)	
Mixed	6 (35.3)	11 (64.7)	
Others	4 (30.8)	9 (69.2)	
FIGO Stage			0.278
I	82 (56.6)	63 (43.4)	
II	27 (42.9)	36 (57.1)	
III	91 (53.8)	78 (46.2)	
IV	11 (45.8)	13 (54.2)	
Tumour Grade			0.947
G1	29 (55.8)	23 (44.2)	
G2	36 (53.7)	31 (46.3)	
G3	131 (53.3)	115 (46.7)	
Surgical Optimal Debulking			0.627
Optimally Debulked	158 (52.3)	144 (47.7)	
Not Optimally Debulked	47 (55.3)	38 (44.7)	
Residual Tumour			0.077
None/Microscopic/<1cm	143 (50.2)	142 (49.8)	
1-2 cm / >2cm	61 (60.4)	40 (39.6)	
Measurable Disease Before ChemoTherapy			0.338
Non- measurable	131 (51.6)	123 (48.4)	
Measurable	66 (55.0)	54 (45.0)	
Platinum sensitivity			0.757
Sensitive	164 (54.5)	137 (45.5)	
Resistant	34 (56.7)	26 (43.3)	

Table S4: LIG4 nuclear protein expression and epithelial ovarian cancers.

Parameter	Low nuc LIG4 N (%)	High nuc LIG4 N(%)	P-Value
Pathological Type			0.785
Serous	142 (67.3)	69 (32.7)	
Mucinous	32 (72.7)	12 (27.3)	
Endometrioid	43 (75.4)	14 (24.6)	
Clear cell carcinoma	21 (72.4)	8 (27.6)	
Mixed	9 (64.3)	5 (35.7)	
Others	7 (58.3)	5 (41.7)	
FIGO Stage			0.971
I	95 (69.9)	41 (30.1)	
II	39 (67.2)	19 (32.8)	
III	106 (70.7)	44 (29.3)	
IV	12 (70.6)	5 (29.4)	
Tumour Grade			0.325
G1	29 (60.4)	19 (39.6)	
G2	42 (68.9)	19 (31.1)	
G3	155 (71.4)	62 (28.6)	
Surgical Optimal Debulking			0.555
Optimally Debulked	195 (69.9)	84 (30.1)	
Not Optimally Debulked	50 (73.5)	18 (26.5)	
Residual Tumour			***0.006***
None/Microscopic/<1cm	177 (67.3)	86 (32.7)	
1-2 cm / >2cm	69 (83.1)	14 (16.9)	
Measurable Disease Before ChemoTherapy			0.371
Non- measurable	161 (68.8)	73 (31.2)	
Measurable	78 (73.6)	28 (26.4)	
Platinum sensitivity			***0.049***
Sensitive	193 (69.7)	84 (30.3)	
Resistant	37 (84.1)	7 (15.9)	

Table S5: Multivariate analyses

Progression Free Survival				
			95.0% CI for Exp(B)	
	Sig.	Exp(B)	Lower	Upper
LIG 1(Nuclear)	***0.001***	1.820	1.285	2.578
LIG 3 (Cyto)	***0.045***	1.409	1.007	1.971
LIG 3 (Nuclear)	0.200	0.796	0.561	1.129
LIG 4(Nuclear)	0.365	0.833	0.561	1.237
**Ovarian Cancer Specific Survival**				
LIG 1(Nuclear)	***0.029***	1.407	1.036	1.910
LIG 3 (Cyto)	0.167	1.237	0.915	1.671
LIG 3 (Nuclear)	0.747	0.950	0.695	1.298
LIG 4(Nuclear)	0.094	0.737	0.516	1.053

Table S6: Genes encoding proteins involved in DNA replication (has03030), base excision repair (hsa03410), nucleotide excision repair (hsa03420) and mismatch repair (hsa03430) KEGG pathways. Genes in bold harbour coding variants in Pt resistant ovarian cancer cell lines.

APEX1	*GTF2H1*	*MPG*	*POLD2*	*RNASEH2B*
APEX2	*GTF2H2*	*MSH2*	*POLD3*	*RNASEH2C*
BIVM-ERCC5	*GTF2H2C*	*MSH3*	*POLD4*	*RPA1*
CCNH	*GTF2H2C_2*	*MSH6*	*POLE*	*RPA2*
CDK7	*GTF2H3*	*MUTYH*	*POLE2*	*RPA3*
CETN2	*GTF2H4*	*NEIL1*	*POLE3*	*RPA4*
CUL4A	*GTF2H5*	*NEIL2*	*POLE4*	*SMUG1*
CUL4B	*HMGB1*	*NEIL3*	*POLL*	*SSBP1*
DDB1	*LIG1*	*NTHL1*	*PRIM1*	*TDG*
DDB2	*LIG3*	*OGG1*	*PRIM2*	*UNG*
DNA2	*MBD4*	*PARP1*	*RAD23A*	*XPA*
ERCC1	*MCM2*	*PARP2*	*RAD23B*	*XPC*
ERCC2	*MCM3*	*PARP3*	*RBX1*	*XRCC1*
ERCC3	*MCM4*	*PARP4*	*RFC1*	
ERCC4	*MCM5*	*PCNA*	*RFC2*	
ERCC5	*MCM6*	*PMS2*	*RFC3*	
ERCC6	*MCM7*	*POLA1*	*RFC4*	
ERCC8	*MLH1*	*POLA2*	*RFC5*	
EXO1	*MLH3*	*POLB*	*RNASEH1*	
FEN1	*MNAT1*	*POLD1*	*RNASEH2A*	

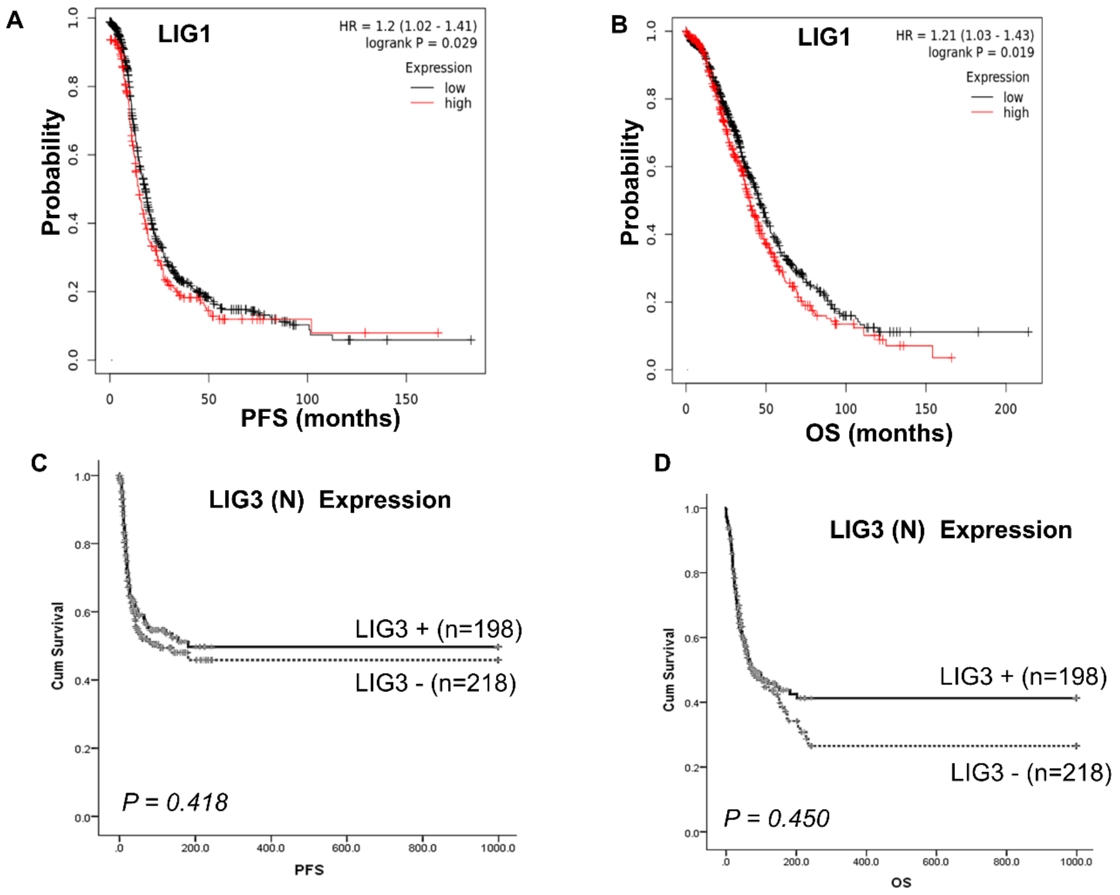

Figure S1: (A) Kaplan Meier curves probability of survival for *LIG1* mRNA expression and PFS. (B) Kaplan Meier curves probability of survival for *LIG1* mRNA expression and OS. (C) Kaplan Meier curves for LIG3 nuclear protein expression and PFS. (D) Kaplan Meier curves for LIG3 nuclear protein expression and OS.

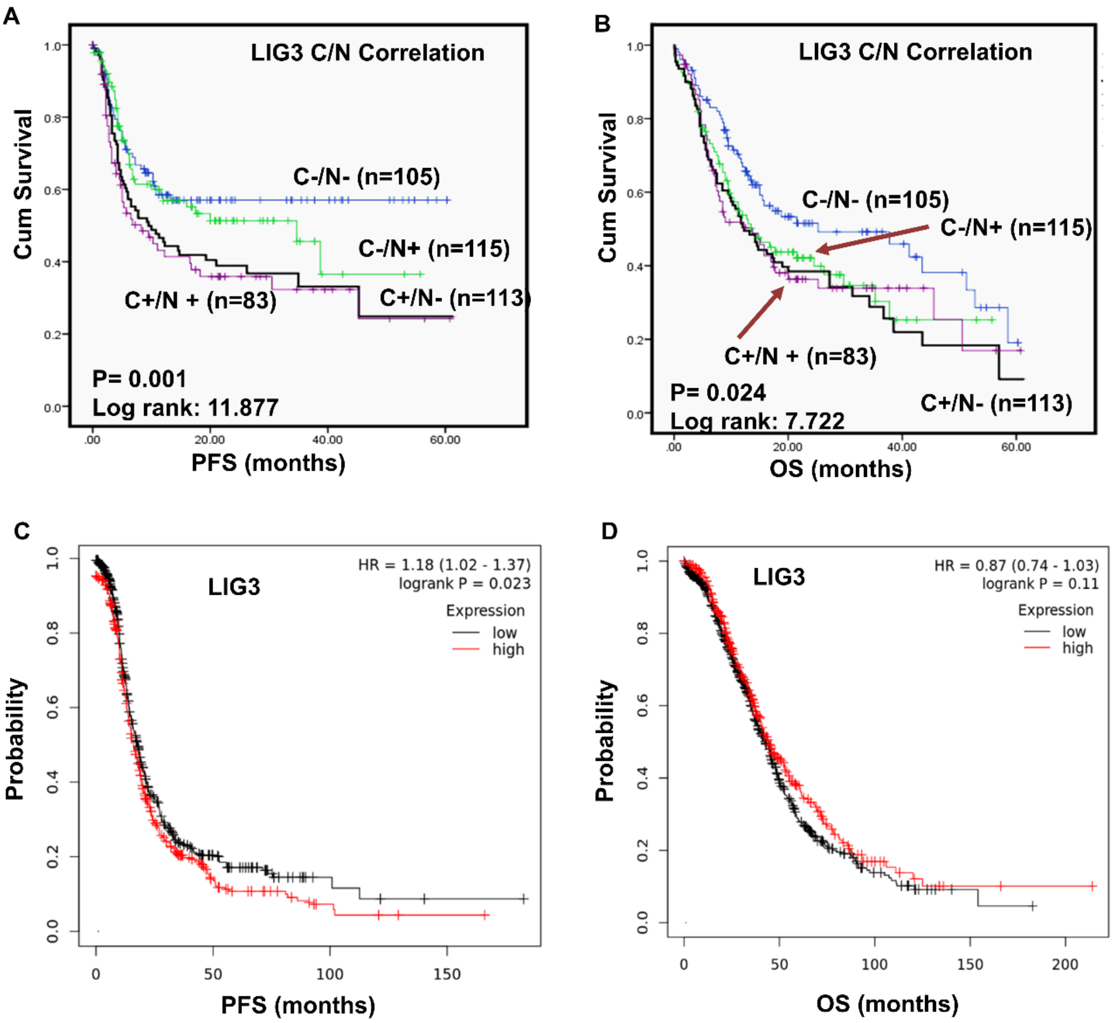

Figure S2: (A) Kaplan Meier curves for LIG3 nuclear and cytoplasmic co-expression and PFS. (**B)** Kaplan Meier curves for LIG3nuclear and cytoplasmic co-expression and overall survival (OS). (**C**) Kaplan Meier curves probability of survival for *LIG3* mRNA expression and PFS. (**D)** Kaplan Meier curves probability of survival for *LIG3* mRNA expression and OS.

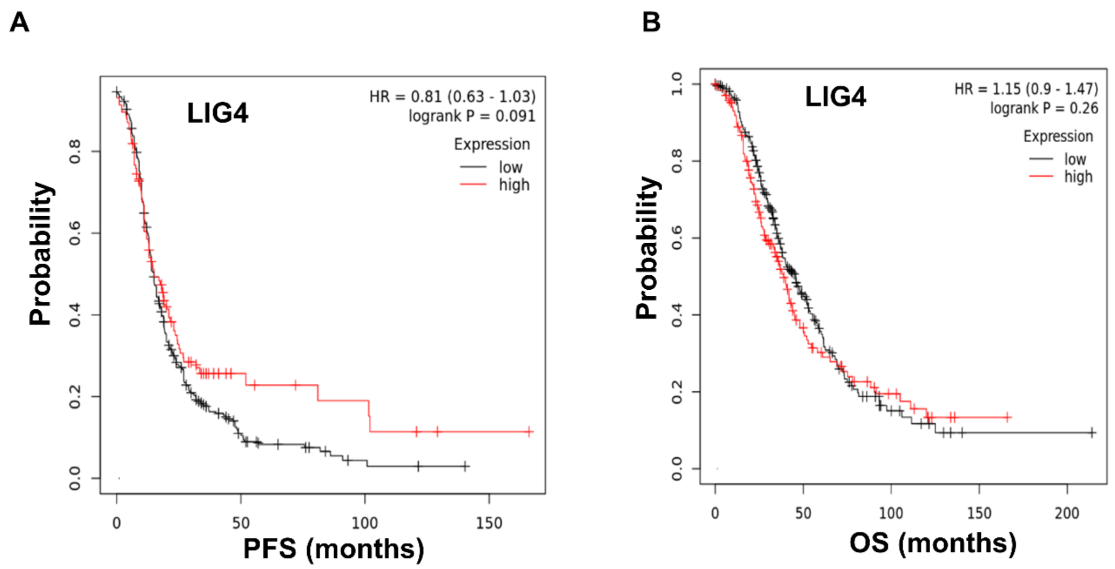

Figure S3: (A) Kaplan Meier curves probability of survival for *LIG4* mRNA expression and PFS. (B) Kaplan Meier curves probability of survival for *LIG4* mRNA expression and OS.

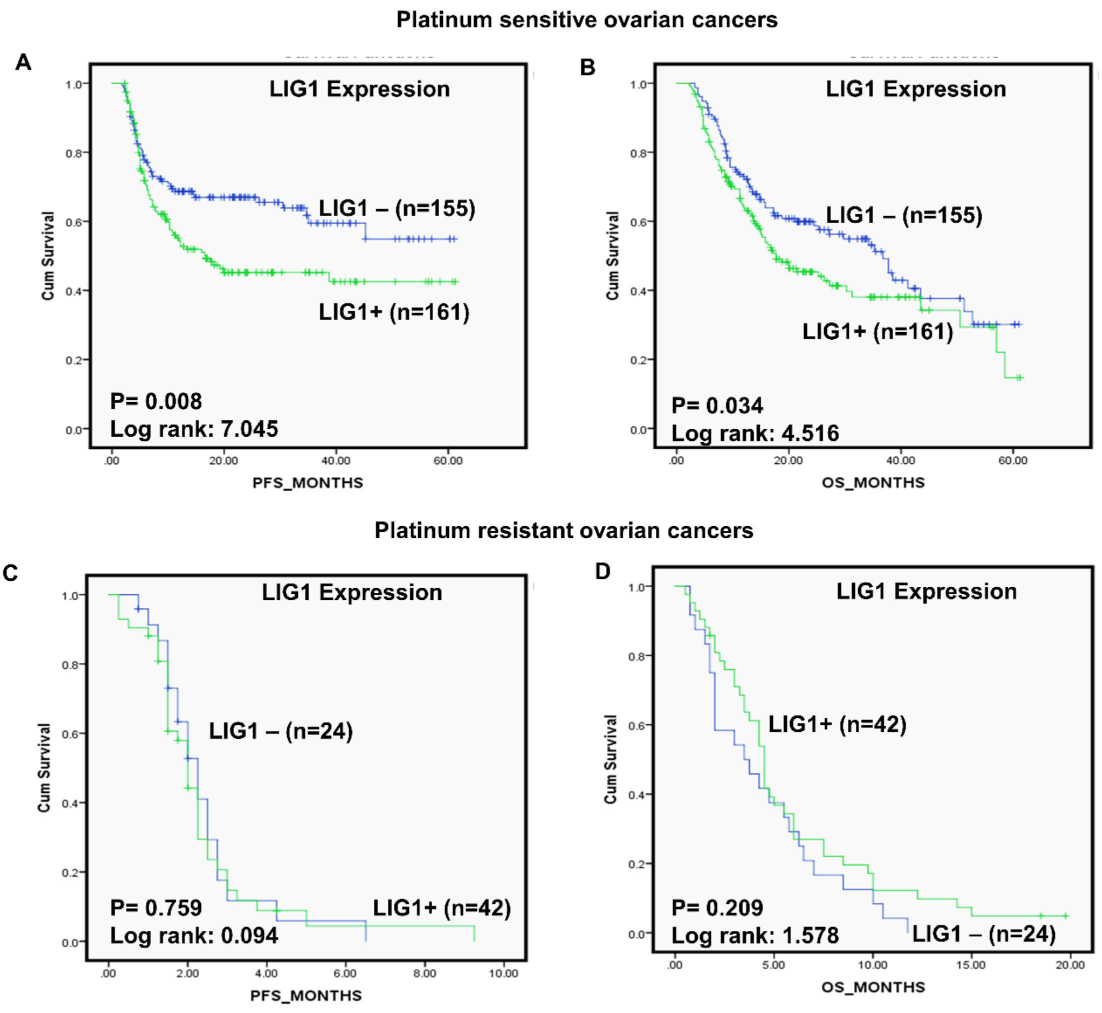

Figure S4: (A) Kaplan Meier curves probability of survival for LIG1 nuclear expression and PFS in platinum sensitive ovarian cancers. (B) Kaplan Meier curves probability of survival for LIG1 nuclear expression and OS in platinum sensitive ovarian cancers. (C) Kaplan Meier curves probability of survival for LIG1 nuclear expression and PFS in platinum resistant ovarian cancers. (B) Kaplan Meier curves probability of survival for LIG1 nuclear expression and OS in platinum resistant ovarian cancers.

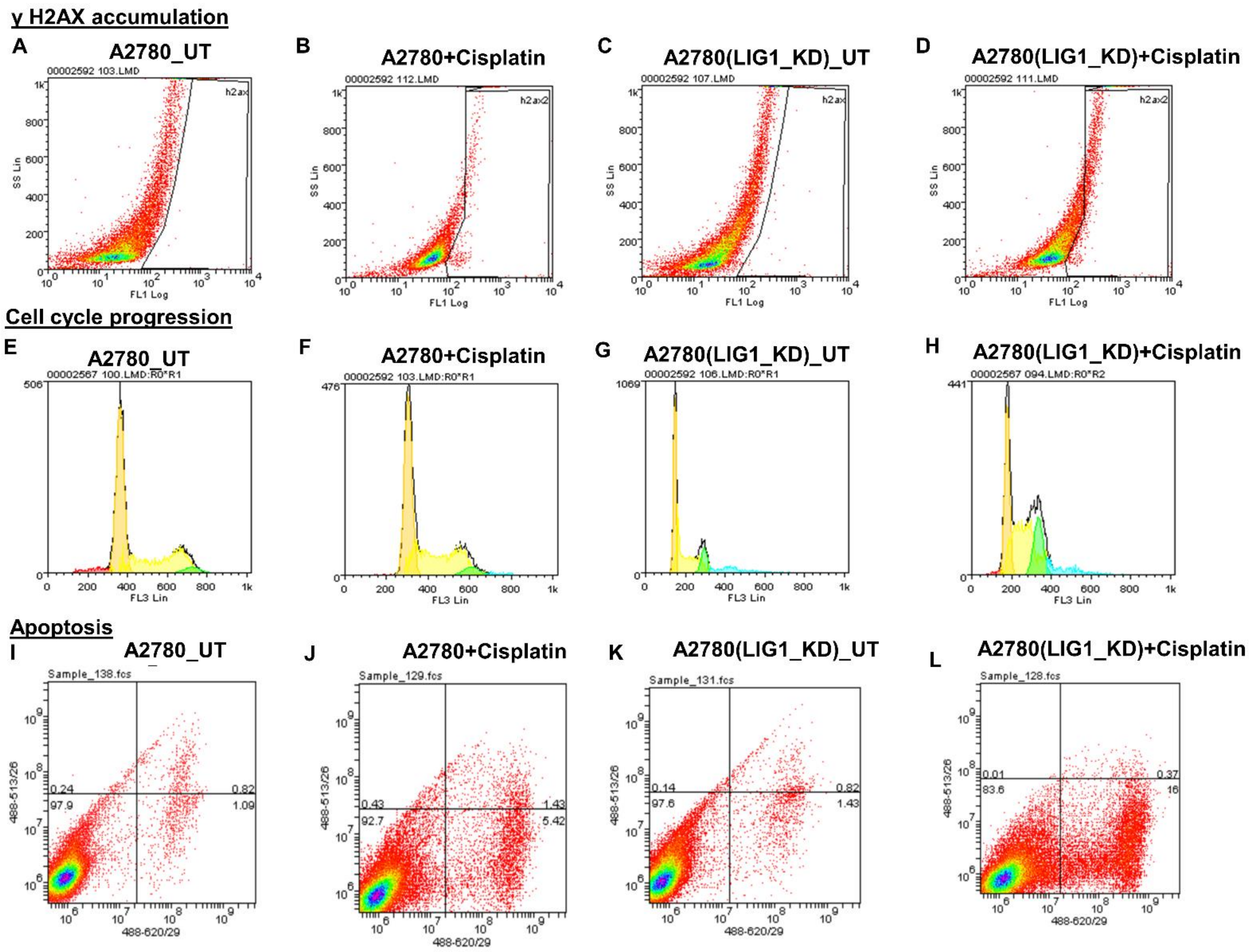

Figure S5: Representative images for γH2AX flow cytometry analysis by weasel software for in: (A) A2780 untreated cells,(B) A2780 treated with Cisplatin, (C) A2780 LIG1_KD untreated and (D) A2780 LIG1_KD treated with cisplatin. Representative images for cell cycle analysis by flow cytometry analysis in weasel software for (E) A2780 untreated cells, (F) A2780 cells treated with Cisplatin, (G) A2780 LIG1_KD untreated and (H) A2780 LIG1_KD treated with cisplatin. Representative images for Annexin V apoptosis analysis by flow cytometry in weasel software in (I) A2780 untreated cells, (J) A2780 cells treated with Cisplatin, (K) A2780 LIG1_KD untreated and (L) A2780 LIG1_KD treated with cisplatin.

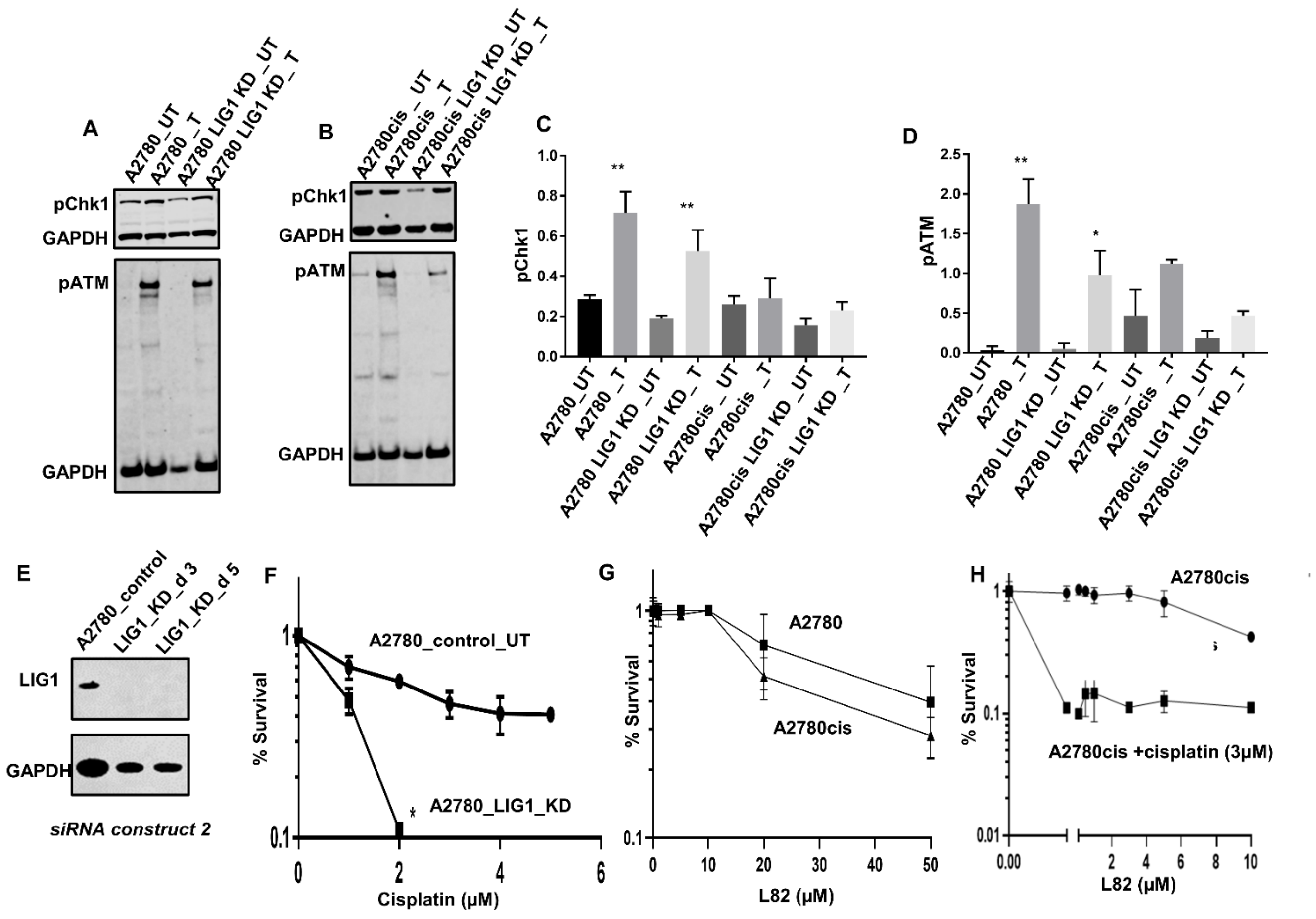

Figure S6: (A) Western blot for pCHK1 and pATM protein levels in A2780 and A2780 LIG1_KD cells untreated and cisplatin treated. (B) Western blot for pCHK1 and pATM protein levels in A2780cis and A2780cis LIG1_KD cells untreated and cisplatin treated. (C) Quantification of pCHK1 protein levels by western blot in A2780 control cells untreated and cisplatin treated, A2780 LIG1_KD untreated and cisplatin treated, A2780cis cells untreated and cisplatin treated A2780cis LIG1_KD cells untreated and cisplatin treated. (C) & (D) Quantification of pCHK1 and pATM protein levels by western blot is shown here. (E) western blot showing LIG1 knock down by siRNA in A2780 cells. Cell lysates have been collected at day3 and day5. (F) Clonogenic survival assay for cisplatin sensitivity in A2780 control and LIG1 knock down cells. (G) Clonogenic survival assay for LG1 inhibitor (L82) sensitivity in

A2780 and A2780cis cells. (H) Clonogenic survival assay forLIG1 inhibitor (L82) in A2780cis cells untreated and pre-treated with cisplatin (3μM).

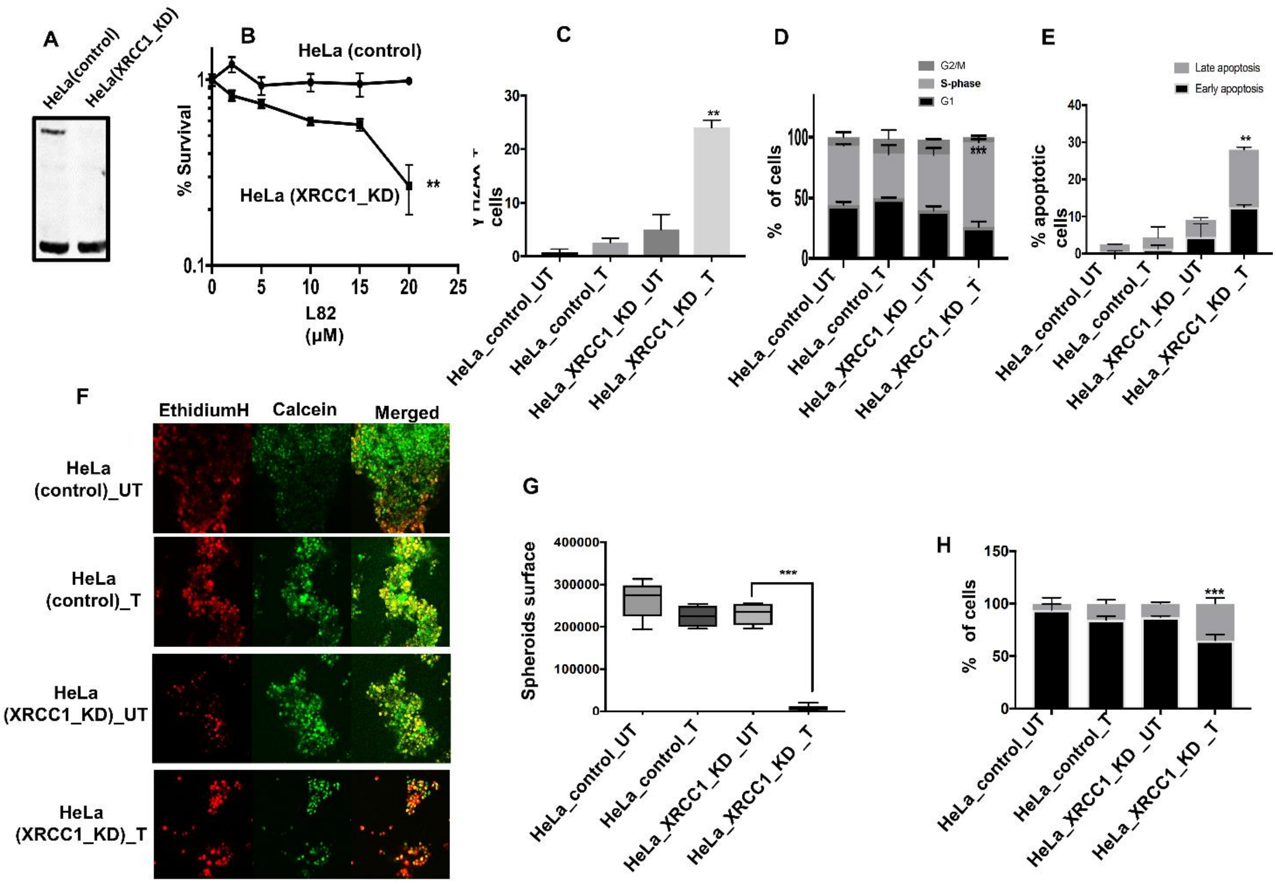

Figure S7: (A) Western blot for LIG1 knock down in Hela cells. (B) Clonogenic survival assay for Ligase inhibitor (L82) in HeLa control and HeLa XRCC1_KD cells. γH2AX analysis by flow cytometry (C), cell cycle analysis by flow cytometry (D) & Annexin V apoptosis analysis (E) in HeLa control and XRCC1_KD cells treated with ligase I inhibitor. (F) Representative photomicrographic images for HeLa control and XRCC1_KD 3D Spheroids treated with LIG1 inhibitor (20μM). (G) Measurement of Spheroids surface area in square pixel by ImageJ software is shown here. (H) Quantification of viable and dead cells is shown here.

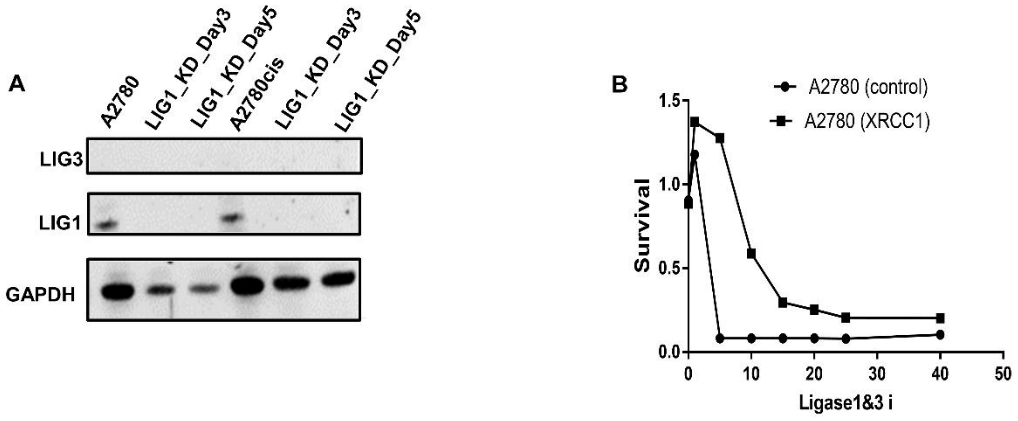

Figure S8: (A)Western blot for LG3 protein levels in A2780 and A2780cis LIG1_KD. Lysates were collected at day3 and day 5. (B) Clonogenic survival assay for LIG1&3 inhibitor in A2780 and A2780 (XRCC1_KD).
